# Comparative genomics and transcriptome analysis reveals potential pathogenic mechanisms of *Microdochium paspali* on seashore paspalum

**DOI:** 10.3389/fmicb.2023.1259241

**Published:** 2023-09-15

**Authors:** Peiyuan Jin, Yixuan Kong, Ze Zhang, Huangwei Zhang, Yinglu Dong, Kurt Lamour, Zhimin Yang, Yuxin Zhou, Jian Hu

**Affiliations:** ^1^College of Agro-grassland Science, Nanjing Agricultural University, Nanjing, China; ^2^Department of Entomology and Plant Pathology, University of Tennessee Institute of Agriculture, Knoxville, TN, United States; ^3^Institute of Botany, Jiangsu Province and Chinese Academy of Sciences, Nanjing, China

**Keywords:** sparse leaf patch, *Microdochium paspali*, seashore paspalum, comparative genomics, comparative transcriptomics analysis

## Abstract

The sparse leaf patch of seashore paspalum (*Paspalum vaginatum* Sw.) caused by *Microdochium paspali* seriously impacts the landscape value of turf and poses a challenge to the maintenance and management of golf courses. Little is known about the genome of *M. paspali* or the potential genes underlying pathogenicity. In this study, we present a high-quality genome assembly of *M. paspali* with 14 contigs using the Nanopore and Illumina platform. The *M. paspali* genome is roughly 37.32 Mb in size and contains 10,365 putative protein-coding genes. These encompass a total of 3,830 pathogen-host interactions (PHI) genes, 481 carbohydrate-active enzymes (CAZymes) coding genes, 105 effectors, and 50 secondary metabolite biosynthetic gene clusters (SMGCs) predicted to be associated with pathogenicity. Comparative genomic analysis suggests *M. paspali* has 672 species-specific genes (SSGs) compared to two previously sequenced non-pathogenic *Microdochium* species, including 24 species-specific gene clusters (SSGCs). Comparative transcriptomic analyses reveal that 739 PHIs, 198 CAZymes, 40 effectors, 21 SMGCs, 213 SSGs, and 4 SSGCs were significantly up-regulated during the process of infection. In conclusion, the study enriches the genomic resources of *Microdochium* species and provides a valuable resource to characterize the pathogenic mechanisms of *M. paspali*.

## 1. Introduction

Seashore paspalum (*Paspalum vaginatum* Sw.), a perennial warm-season grass with dense rhizomes and stolon’s, is widely distributed in Malaysia, Australia, South Africa, Southeastern China, Southern United States and other tropical and subtropical coastal areas (Lonard et al., 2015). It is one of the most attractive grass species because of its excellent characteristics, including trampling resistance, salt tolerance, drought tolerance, and is widely used in forage production, ecological restoration, landscape, and athletic fields (e. g. golf courses) (Pompeiano et al., 2016; Karimi et al., 2018). However, with its increasing use, the disease problems of seashore paspalum have become severe, and many fungal diseases have been reported to cause infection, including fusarium blight (*Fusarium* sp.), large patch (*Rhizoctonia solani*), dollar spot (*Clarireedia* spp.), basal leaf blight (*Waitea circinata*), and take-all patch (*Gaeumannomyces graminis* var. *graminis*) (Kammerer et al., 2011; Wang, 2015; Zhang et al., 2022).

**Figure 1:**
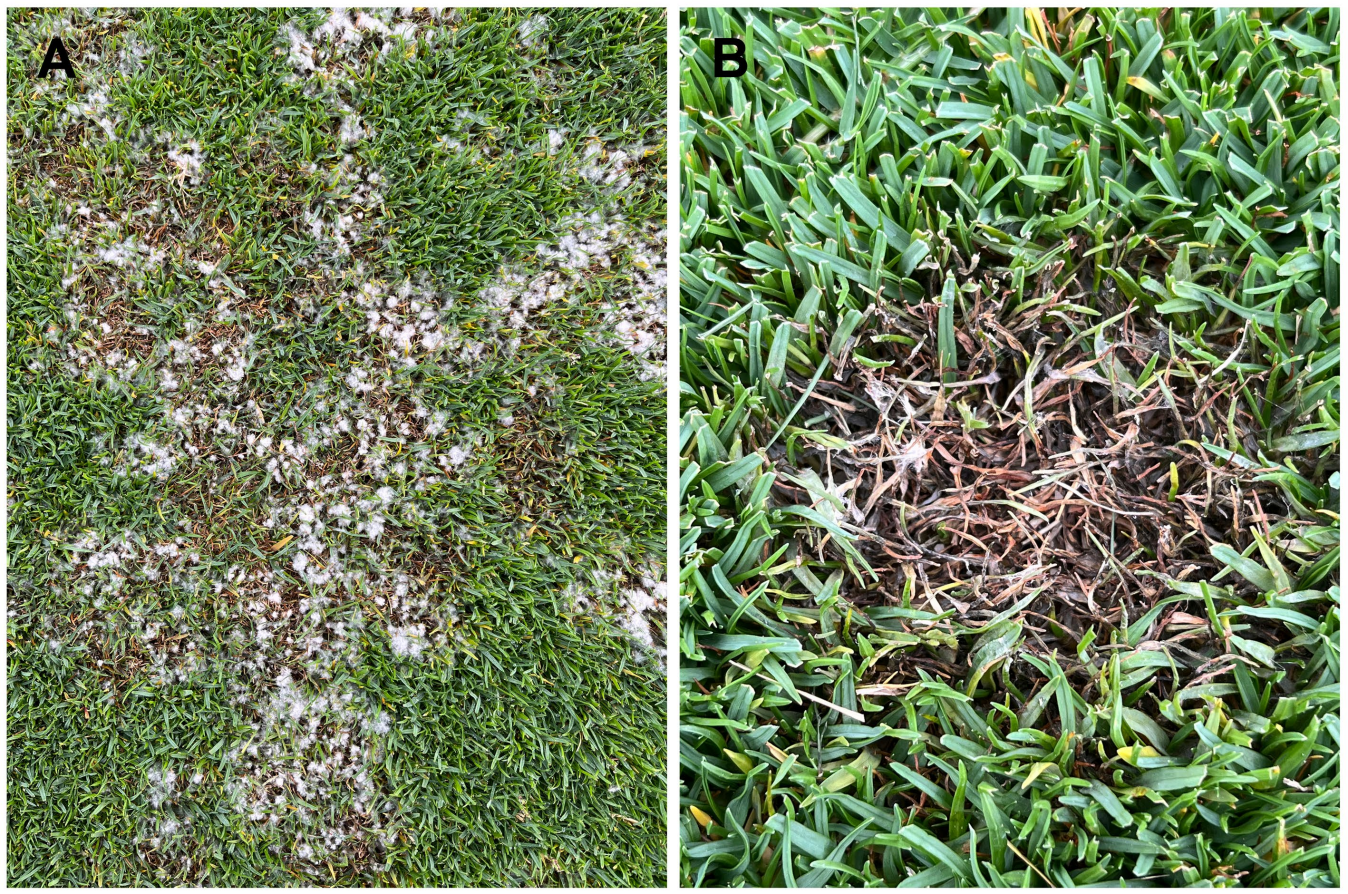
Symptoms of sparse leaf patch on seashore paspalum caused by *Microdochium paspali.* **(A)** The irregular sparse patches on the diseased seashore paspalum fairway. **(B)** Infected leaves died quickly, with white mycelium attached.

In 2015, a new disease of seashore paspalum named “sparse leaf patch” was reported at golf courses in Hainan province, China. The disease is caused by a new plant pathogenic fungal species, *Microdochium paspali* (Zhang et al., 2017). At present, there is no report that *M. paspali* has been isolated from other turfgrasses or plants, but study has verified that it can infect other warm-season and cold-season turfgrasses in control environments (Zhang et al., 2017). Initially, the disease symptoms of infected seashore paspalum are dark green spots with sizes varying from 3–10 cm in diameter. The spots are white with clumped mycelia when the environment is highly humid, especially in the morning. The infected leaves show signs of waterlogging. As the disease progresses, the leaves turn yellow and may become necrotic. And finally, the whole leaves can die rapidly leading to obviously sparse patches on the turf; seriously impacting the landscape and causing significant economic losses (Figure 1).

*Microdochium* is a genus in Microdochiaceae (Xylariales), first described by Sydow in Germany with *M. phragmitis* isolated from reed leaves as the model. It is distributed worldwide, especially in Asia and Europe (Huang et al., 2020). Many *Microdochium* species are important plant pathogens causing diseases of cereals and grasses with severe yield losses. In addition to *M. paspali*, *M. majus*, and *M. nivale* are reported to cause pink snow mold of cereals and cool-season turfgrass in winter (Jewell and Hsiang, 2013). *M. oryzae* can cause leaf scale of rice (*Oryza sativa*) (Parkinson, 1980), *M. maydis* can cause tar-spot disease of maize (*Zea mays*) (Müller and Samuels, 1984), and recently, *M. poae* was reported to cause leaf blight on Kentucky bluegrass (*Poa pratensis*) and creeping bentgrass (*Agrostis stolonifera*) in North China (Liang et al., 2019). Some *Microdochium* species also have biological control effects. For example, the spores of the endophyte *M. bolleyi* can effectively inhibit the take-all disease of wheat and barley (Jadubansa et al., 1994).

At present, studies on *Microdochium* species mainly focused on the identification and biological characterization of the pathogenic or endophytic fungi, but there is a lack of genome-scale studies. In the NCBI database, only a few gene sequences commonly used for fungal classifications have been recorded for *Microdochium* species, and the genomes of only two endophytes, *M. bolleyi* (David et al., 2016) and *M. trichocladiopsis* (Mesny et al., 2021), have been sequenced. Sparse leaf patch caused by *M. paspali* has become one of the most serious diseases on seashore paspalum at golf courses in East and South China. Current research on *M. paspali* is limited and little is known at molecular level (Zhang et al., 2017). In recent years, comparative genomics and transcriptomics have been successfully applied to plant pathogenic fungi (Li et al., 2009; Shen et al., 2017) to better understand the mechanisms of species evolution, pathogenicity and pathogen-host interactions.

In this study, we sequenced the genome of *M. paspali* using a combination of Nanopore and Illumina high-throughput sequencing technologies. The putative pathogenic related factors in *M. paspali* were systematically predicted, including pathogen-host interaction (PHI) proteins, carbohydrate-active enzymes (CAZymes), secreted proteins and effector proteins, species-specific genes (SSGs). The species-specific secondary metabolite biosynthetic gene clusters (SMGCs) were further analyzed by comparative genomics with the non-pathogenic fungi, *M. bolleyi* and *M. trichocladiopsis*. In addition, comparative transcriptomics analysis were performed to identify the putative disease-related factors during the infection of *M. paspali* on seashore paspalum. This study enriches the genome resources of *Microdochium* species and provides a scientific basis for further characterization of the mechanisms of pathogenicity and pathogen-host interactions.

## 2. Materials and methods

### 2.1. Genomic DNA extraction

The *M. paspali* strain I16 was preserved at the College of Agro-grassland Science, Nanjing Agricultural University. It was isolated from symptomatic leaves of seashore paspalum (*Paspalum vaginatum*) at the research station of the Jurong Turf Institute. The strain, stored in glycerol (20%), was inoculated on potato dextrose agar (PDA) medium, and incubated for 3 d at 25°C in the dark. Thereafter, actively growing mycelial plugs were transferred to fresh PDA at 25°C for 7 d, and subsequently the mycelia were collected. Highly purified total genomic DNA was extracted according to a modified CTAB method (Murray and Thompson, 1980). DNA quality and concentration were examined by electrophoresis in 1% agarose gels (BIOWEST Regular agarose G-10) and the Nanodrop ND-100 (Nanodrop Technologies, Waltham, MA, United States), and sent to Wuhan Benagen Tech Solutions Co., Ltd. for genome sequencing.

### 2.2. Genome sequencing and assembly

The whole-genome of *M. paspali* was sequenced by the third generation nanopore sequencing combined with the second generation Illumina sequencing. For nanopore sequencing, the Oxford Nanopore Technology (ONT) library was constructed following the manufacturer’s protocols of a Ligation Sequencing Kit SQK-LSK109 and a Native Barcoding Expansion Pack EXP-NBD104/114 (Oxford Nanopore Technologies, Oxford, UK). The prepared ONT library was loaded onto flow cells for single molecule sequencing on a PromethION device (Oxford Nanopore Technologies). Illumina sequencing was conducted on an Illumina NovaSeq (Illumina Inc., San Diego, CA, United States) after the genomic DNA was randomly fragmented by Covaris M220 Focused Ultra-sonicator device (Covaris, LLC, United States). The library for Illumina sequencing was constructed following the steps of DNA fragment end repair, 3′ A-tailing, adapter ligation, purification, and PCR amplification. The raw sequencing data were filtered to obtain clean reads, by removing reads containing adapters and ploy-N and low-quality reads with more than 50% of the bases having Q-scores ≤10 in the whole read. The clean reads were analyzed by Genomescope v1.0 (Vurture et al., 2017) with k-mer (*k* = 19) to estimate the genome size, heterozygosity, and duplication rate before assembly. An initial splicing result was obtained by performing genome error correction and splicing with NECAT software (Chen et al., 2021). The obtained result was subjected to two rounds of error correction based on the third generation sequencing data using Racon v1.4.11 (Vaser et al., 2017), followed by two rounds of Pilon v1.23 (Walker et al., 2014) error correction of the second generation reads. Finally, the haplotigs (also known as duplicate regions) were removed using Purge Haplotigs (Roach et al., 2018) with default parameters. The completeness of the genome assembly was assessed based on the lineage data set fungi_odb10 using BUSCO v4.1.4 (Simão et al., 2015).

### 2.3. Genome structural prediction and functional annotation

The *de novo* prediction of coding genes in the *M. paspali* genome was mainly performed with BRAKER v2.1.4 (Brůna et al., 2021), a fully automated pipeline for gene predictions with GeneMark-EP+ and AUGUSTUS. Firstly, the genomic data and RNA-seq data were used for obtaining initial gene structure using the GeneMark-EP+ tool, and then AUGUSTUS was used for further training based on the initial gene structure to generate the final gene prediction results. Non-coding RNA was predicted and statistically classified based on the Rfam database using Infernal v1.1.3 (Nawrocki and Eddy, 2013). The RepeatModeler v2.0 (Flynn et al., 2020) was used to construct a *de novo* repeat library. The repbase libraries (Bao et al., 2015) were merged and RepeatMasker v4.0.5 (Tempel, 2012) was used for repeat sequence annotations at the genome level.

Functional annotations of coding genes in the *M. paspali* genome were performed based on searches of sequence and motif similarities. The protein sequences encoded by the genes were compared with the existing protein databases Uniprot, Gene Ontology (GO), Kyoto Encyclopedia of Genes and Genomes (KEGG), Clusters of Orthologous Groups of Proteins (COG), Nonredundant (Nr), Pathway, and Reference Sequence (Refseq) with BLASTP (BLAST+ v2.9, *E*-value <1e-5) (Camacho et al., 2009) to predict gene function. The prediction of structural domains was performed using the program HMMScan in HMMER v3.2.1 (Johnson et al., 2010), and the conserved sequences, motifs and structural domains of the proteins were obtained The predicted proteins were further annotated using the Protein Families (Pfam) database (Mistry et al., 2021) and InterProScan v5.0 (Jones et al., 2014).

### 2.4. Comparative genomics analysis

The genome sequences of *Microdochium bolleyi* (accession no. GCA_001566295.1) and *Microdochium trichocladiopsis* (accession no. GCA_020744255.1) were selected as the reference genomes of homologous species for *M. paspali*. The genomes of *Fusarium graminearum* (accession no. GCA_900044135.1), *Fusarium poae* (accession no. GCA_019609905.1), *Fusarium equiseti* (accession no. GCA_910393935.1), *Fusarium solani* (accession no. GCA_020744495.1), *Fusarium oxysporum* (accession no. GCA_013085055.1), *Fusarium verticillioides* (accession no. GCA_000149555.1), *Clarireedia jacksonii* (accession no. GCA_026225895.1), *Magnaporthiopsis poae* (accession no. GCA_000193285.1), *Botrytis cinerea* (accession no. GCA_000143535.4), *Sclerotinia sclerotiorum* (accession no. GCA_001857865.1), and *Truncatella angustata* (accession no. GCA_020726525.1), were selected for comparisons with the genomic data of *M. paspali*. Ortholog clustering analysis was performed using OrthoFinder v2.3.12 (Emms and Kelly, 2019) based on all the protein sequences from the selected fungal species. MUSCLE v3.8.31 (Edgar, 2004) was used to perform multi-sequence alignments based on the protein sequences of each single-copy gene family identified by clustering and trimAL v1.4 was used to filter the alignment results. Finally, the filtered comparison results were combined and a phylogenetic tree of these species constructed using RAXML v8.2.10 (Stamatakis, 2014).

Subsequently, the protein-coding genes of *M. paspali* with identity or coverage of <50% in *M. bolleyi* and *M. trichocladiopsis* genomes were screened by BLASTP (*E*-value <1e-5), and these genes were integrated with the results of unique orthologous groups and classified as SSGs of *M. paspali*. Gene sequences in the genomes of the three *Microdochium* species were compared using DIAMOND v0.9.29 (*E*-value <1e-5, C score > 0.5) (Buchfink et al., 2015) to identify similar gene pairs. MCScanX-jcvi (*E*-value <1e-5) (Wang et al., 2012) was further used to determine whether similar gene pairs were adjacent on the contigs. Eventually, genes in all the collinear blocks were obtained and the genome collinearity among the three *Microdochium* species was visualized.

### 2.5. Transcriptome sequencing and analyses

To obtain the interactive transcriptomes between *M. paspali* and seashore paspalum, both fungal mycelia and plant leaf tissues were collected after inoculation. Specifically, ten actively growing mycelial plugs (5 mm in diameter) of *M. paspali* were put into potato dextrose broth (PDB) medium and grown for 7 days. Mycelia were collected and rinsed with sterile distilled water (SDW) and a portion was stored directly in centrifuge tubes in liquid nitrogen. This served as the control (0 h of inoculation) with the rest being used to make a mycelial suspension by adding SDW and pulverizing with a grinder. The mycelial suspension was evenly sprayed on healthy seashore paspalum cultivar “Sea Spray” until water drops fell off the leaves. The plants were then transferred to a sterilized glass box and placed in a greenhouse at 25°C. Inoculated plants were watered daily to maintain high humidity for infection. It was observed that the mycelium of *M. paspali* began to grow gradually within 0–12 h after infection, and there were no visible symptoms on the leaves. At 24 h post infection, the water-soaked leaves could be observed, and at 36 h the symptoms of the leaves began to worsen and chlorosis was observed. At 48 h post infection the mycelium of *M. paspali* spread all over the leaves, which appeared to be uniformly blighted and showed obvious black lesions. Therefore, we collected leaf tissues at 6 h, 12 h, 24 h, 36 h, and 48 h after inoculation (Supplementary Figure S1), and preserved in liquid nitrogen before sequencing. Three biological replicates for each treatment were harvested.

RNA of the samples was extracted using the TRlzol Reagent (Life technologies, California, United States) and RNA concentrations and purity were measured using the NanoDrop 2000 (Thermo Fisher Scientific, Wilmington, DE). RNA integrity was assessed using the RNA Nano 6,000 Assay Kit and the Agilent Bioanalyzer 2,100 system (Agilent Technologies, CA, United States). RNA-seq was performed using the Illumina NovaSeq6000 platform and the 150 bp paired-end chemistries. Raw reads were processed through in-house perl scripts to obtain clean reads by removing reads containing adapters and ploy-N and low-quality reads with more than 50% of the bases having Q-scores ≤10 in the whole read. Subsequently, Hisat2 v2.2.1 (Kim et al., 2015) was used to map the clean reads to the genome of *M. paspali*. Fragments per kilobase of transcript per million fragments mapped (FPKM) was applied to measure the expression level of a gene or transcript by StringTie (Pertea et al., 2015) employing the maximum flow algorithm. DESeq2 v1.30.1 (Love et al., 2014) provided statistical routines for determining the differential expressions in digital gene expression data using a model based on the negative binomial distribution. Genes with an adjusted fold change ≥1.5 and false discovery rate (FDR) <0.01 were assigned as differentially expressed. Principal component analysis (PCA) of different samples and GO enrichment analysis of differential expressed genes (DEGs) were performed using TBtools v1.108 (Chen et al., 2020).

### 2.6. Prediction and analysis of pathogenicity-related genes

Homologs of known PHI genes were annotated using the BLASTP (*E*-value <1e-5) based on the PHI v4.14 database (Urban et al., 2021). The prediction and annotation of CAZymes in *M. paspali* was performed with the dbCAN2 meta-server (*E*-value <1e-5, coverage >35%) (Zhang et al., 2018). Identification of SMGCs was accomplished in the three species of *Microdochium* and comparisons with known clusters using antiSMASH v6.0 (Blin et al., 2021) at default parameters.

The classical secreted proteins in *M. paspali* were identified according to multiple software analyses. The protein sequences of all genes were analyzed using SignalP v5.0 (Almagro Armenteros et al., 2019a,b) to identify proteins containing signal peptides. TMHMM v2.0 (Krogh et al., 2001) was used to eliminate proteins containing transmembrane domains. PredGPI (Pierleoni et al., 2008) was used to eliminate GPI ankyrin. Subcellular localization analysis was performed by TargetP v2.0 (Almagro Armenteros et al., 2019a,b) and the exocrine secreted proteins were screened as classical secreted proteins. Finally, EffecorP v3.0 (Sperschneider and Dodds, 2022) was used for further prediction, and proteins with amino acid length < 300 and the number of cysteine residues ≥4 (Jones et al., 2018) were manually screened as putative effectors.

The density statistics of each type of pathogenicity-related genes and transposable elements in the *M. paspali* genome were performed at 50-kb length intervals using TBtools v1.108 (Chen et al., 2020). Based on the gene density profile, the circos plot of *M. paspali* genome was performed using shinyCircos v2.0 (Wang et al., 2023) to display the genome-wide pathogenicity-related genes density distribution.

## 3. Results

### 3.1. High-quality genome assembly and annotation of *Microdochium paspali*

The genome of *M. paspali* was assembled using a combination of Nanopore sequencing and correction with Illumina short reads. Nanopore sequencing constituted 5.5 Gb data, a total of 707,581 nanopore long reads with an average length of 7.4 kb were obtained after data filtering. Additionally, approximately 28.73 million Illumina short reads were used to correct errors. The assembled genome size of 37.32 Mb was 94.29% of the estimated genome size (39.58 Mb) by K-mer analysis (Supplementary Figure S2; Supplementary Table S1). The genome consisted of 14 contigs with an N50 of 3.54 Mb. The maximum contig length was 5,367,041 bp (Contig 1), the minimum contig length was 102,989 bp (Contig 14), and the average length was 2,665,914 bp. The GC content of the assembled contigs was 54.17% (Table 1). Using the fungi_odb10 data set of BUSCO to evaluate the integrity of genome assembly; 99.1% of the expected single-copy orthologs (758) were present in the *M. paspali* genome (Table 1), indicating a high quality genome. Compared with other sequenced species in the *Microdochium* genus, the size of the *M. paspali* assembly was smaller than those in *M. trichocladiopsis* (49.28 Mb) and *M. bolleyi* (38.84 Mb) (David et al., 2016; Mesny et al., 2021).

**Table 1:** Features of the *Microdochium paspali* genome assembly and annotations.

Features	*Microdochium paspali*
Genome assembly size (bp)	37,322,795
Number of contigs	14
N50 contig (bp)	3,540,938
Maximum contig length (bp)	5,367,041
Minimum contig length (bp)	102,989
Average contig length (bp)	2,665,913.93
GC content (%)	54.17
Number of complete BUSCOs	751
Number of fragmented BUSCOs	0
Number of missing BUSCOs	7
Number of coding genes	10,365
Average gene length (bp)	1,703.35
Total gene length (bp)	17,655,223
Average exon number per gene	2.71
Repeat sequence (%)	1.77
Number of rRNAs	40
Number of sRNAs	3
Number of snRNAs	25
Number of tRNAs	137

A total of 10,365 protein-coding genes were predicted in the genome of *M. paspali* with an average gene length of 1703 bp and an average of 2.71 exons per gene, while no gene was predicted on contig 14. The total length of genes were 17.66 Mb, accounting for 47.32% of the genome (Table 1). The repeat sequences identified in *M. paspali* represented 1.78% of the genome length. Most of the repeated sequences were simple repeats with a coverage of 1.13%, while the transposable elements (long terminal repeats (LTR), Long interspersed nuclear elements (LINE), short interspersed nuclear elements (SINE), and DNA element) were 0.51%% (Supplementary Table S2). In addition, a total of 205 non-coding RNAs were annotated, including 40 rRNAs, 3 sRNAs, 25 snRNAs, and 137 tRNAs (Table 1).

To predict gene function, functional annotation was performed using several public databases including GO, KEGG, eggCOG, Nr, Uniprot, Pfam, Pathway, Refseq, and Interproscan. There were 9,877 genes annotated, accounting for 95.29% of the total predicted genes (Supplementary Table S3). The GO database annotated 5,839 genes (56.33%), and most genes involved in the biological processes were transmembrane transport and protein transport; the cellular components were mainly concentrated in nucleus, cytoplasm, and integral component of membrane; the molecular functions were mainly concentrated in binding, oxidoreductase activity, and monooxygenase activity (Supplementary Figure S3). There were 2,405 genes (23.20%) annotated in the KEGG database, of which, 2,263 genes (21.83%) were enriched into 331 metabolic pathways, including amino acid metabolism, carbohydrate metabolism, global and overview maps (Supplementary Figure S4). The COG analysis showed that 810 genes (7.81%) were annotated and clustered into 24 orthologous groups. The functions of these genes were mainly concentrated in carbohydrate transport and metabolism (97 genes), lipid transport and metabolism (87 genes), amino acid transport and metabolism (79 genes), general function prediction only (76 genes), translation, ribosomal structure and biogenesis (66 genes), and energy production and conversion (63 genes) (Supplementary Figure S5).

### 3.2. Identification of pathogenicity-related genes in *Microdochium paspali*

The pathogenicity-related genes in *M. paspali* were systematically identified by a combination of bioinformatic prediction and transcriptome sequencing. After sequencing quality control, a total of 198.78 Gb of clean data was obtained for 18 samples through transcriptome sequencing, and the clean data of each sample reached 10.03 Gb. The samples generated clean reads of approximately 33.48 M to 41.86 M, the percentages of Q30 bases were all higher than 91.68%, and a total of 9,629 genes (92.90% of the genes in *M. paspali* genome) were detected in all samples (Supplementary Table S4). The result of PCA showed that the samples at each time point after infection were obviously separated from the samples before infection (Supplementary Figure S6). A total of 1,983 (1,109 up-regulated and 874 down-regulated), 2,535 (1,487 up-regulated and 1,048 down-regulated), 3,244 (1867 up-regulated and 1,377 down-regulated), 3,700 (1908 up-regulated and 1792 down-regulated), and 4,157 (2,338 up-regulated and 1819 down-regulated) DEGs were identified at 6 h, 12 h, 24 h, 36 h, and 48 h after infection, respectively (Supplementary Figure S7; Supplementary Table S5). Subsequently, we performed GO analysis of the DEGs at each time after *M. paspali* infection. The result showed that the up-regulated DEGs functions were significantly enriched in catalytic activity, membrane and metabolic process, while the down-regulated DEGs functions were significantly associated with binding, metabolic process and cellular process (Supplementary Figure S8).

**Figure 2:**
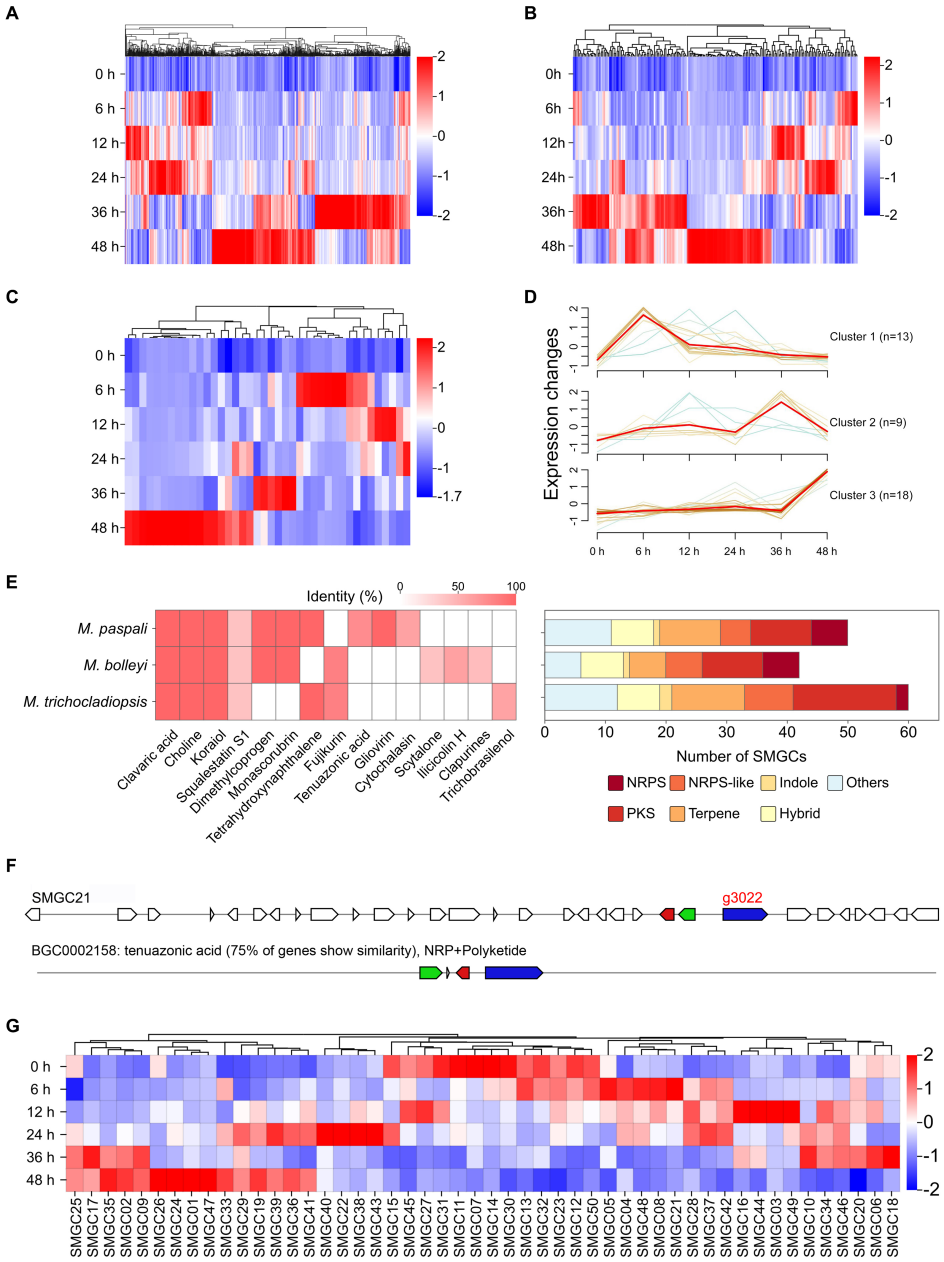
Identification and analysis of pathogenicity-related genes in *Microdochium paspali*. **(A–C)** Heat map of the normalized fragments per kilobase of transcript per million fragments mapped (FPKM) values of up-regulated pathogenicity-related pathogen-host interactions (PHI) genes, carbohydrate active enzymes (CAZymes) genes, and effector genes at different infection stages. The expression level was indicated by different colors, red indicates a high expression and blue indicates a low expression. **(D)** Expression patterns of up-regulated expressed effector genes during the infection process. **(E)** The presence of predicted secondary metabolite biosynthetic gene clusters (SMGCs) in the three *Microdochium* species compared to known gene clusters and the distribution of the SMGC core biosynthetic genes family in each species. NRPS: nonribosomal peptide synthetase, NRPS-like: NRPS-like fragment, PKS: poly keytide synthase, HYBRID: multiple core biosynthetic genes. **(F)** Comparison of the similarity between *M. paspali* SMGC21 and the tenuazonic acid biosynthetic gene cluster from *Pyricularia oryzae 70–15*.0 **(G)** Heat map of normalized FPKM values of SMGC core biosynthetic genes at different infection stages.

Abundant pathogenicity-related genes were found in *M. paspali* genome, a total of 3,830 genes were annotated, accounting for 29.72% of the total predicted genes by using the PHI database (Supplementary Table S6). Of these genes, 1,819, 342 and 136 were annotated as related to reduced virulence, loss of pathogenicity, and increased virulence, respectively, and 739 pathogenicity-related PHI homologs were up-regulated during infection (Figure 2A). Most of the annotated genes were homologs from *Fusarium graminearum, Magnaporthe oryzae,* and *Aspergillus fumigatus* (Supplementary Table S6).

In the *M. paspali* genome, 481 CAZymes coding genes were predicted suggesting that *M. paspali* may use a variety of CAZymes to break through the first barrier of host cell wall during infection (Supplementary Table S7). The glycoside hydrolases (GHs) accounted for the largest number (247), followed by 102 auxiliary activities (AAs), 85 glycosyl transferases (GTs), 37 carbohydrate esterases (CEs), 13 carbohydrate-binding modules (CBMs), and 9 polysaccharide lyases (PLs). There were 12 genes with two CAZymes annotations (1 AA/CBM and 11 GH/CBM). In total, one hundred ninety-eight CAZymes in *M. paspali* were up-regulated in at least one time point after infection (Figure 2B).

A total of 678 secreted proteins were predicted in *M. paspali*, of which 105 were putative effectors. Among these putative effectors, fifty-six were hypothetical proteins of unknown function, and thirty-one were not annotated in the NR database. Thirty-two putative effectors shared homologies with the functional domains, and ten were related to reduced virulence, two were related to increased virulence, one was related to loss of pathogenicity, and four were clearly annotated as effectors in other pathogenic fungi (Supplementary Table S8). Transcriptome analysis revealed that forty of the putative effectors were significantly up-regulated during *M. paspali* infection (Figure 2C), and the expression patterns of up-regulated genes encoding effectors were clustered into three groups, where 13, 9, and 18 putative effectors showed significantly dynamic changes in the early (6–24 h), middle (36 h), and late (48 h) stage of the infection, respectively (Figure 2D). Six putative effectors (g1621, g1879, g2155, g3289, g421, and g9387) were up-regulated at all infection stages.

In order to evaluate the production potential of secondary metabolites of *M*. *paspali*, SMGCs were further predicted, and a total of 50 SMGCs were identified in *M*. *paspali* (Supplementary Table S9). Three unique SMGCs (SMGC 21, SMGC 6 and SMGC 39) in *M*. *paspali* were identified to be involved in the synthesis of known compounds, as compared with *M. bolleyi* and *M. trichocladiopsis* (Figure 2E). The similarity between SMGC 21 and the tenuazonic acid (TeA) biosynthetic gene cluster from *Pyricularia oryzae* reached 75% (Figure 2F), which is a mycotoxin widely produced in various plant pathogenic fungi and causes significant damage to a variety of crops (Lebrun et al., 1988). Transcriptome analysis revealed that twenty-one SMGCs were up-regulated after infection (Figure 2G) and SMGC 21 was specifically up-regulated at the early stage of infection (6–24 h).

### 3.3. Identification of species-specific genes in *Microdochium paspali*

**Figure 3:**
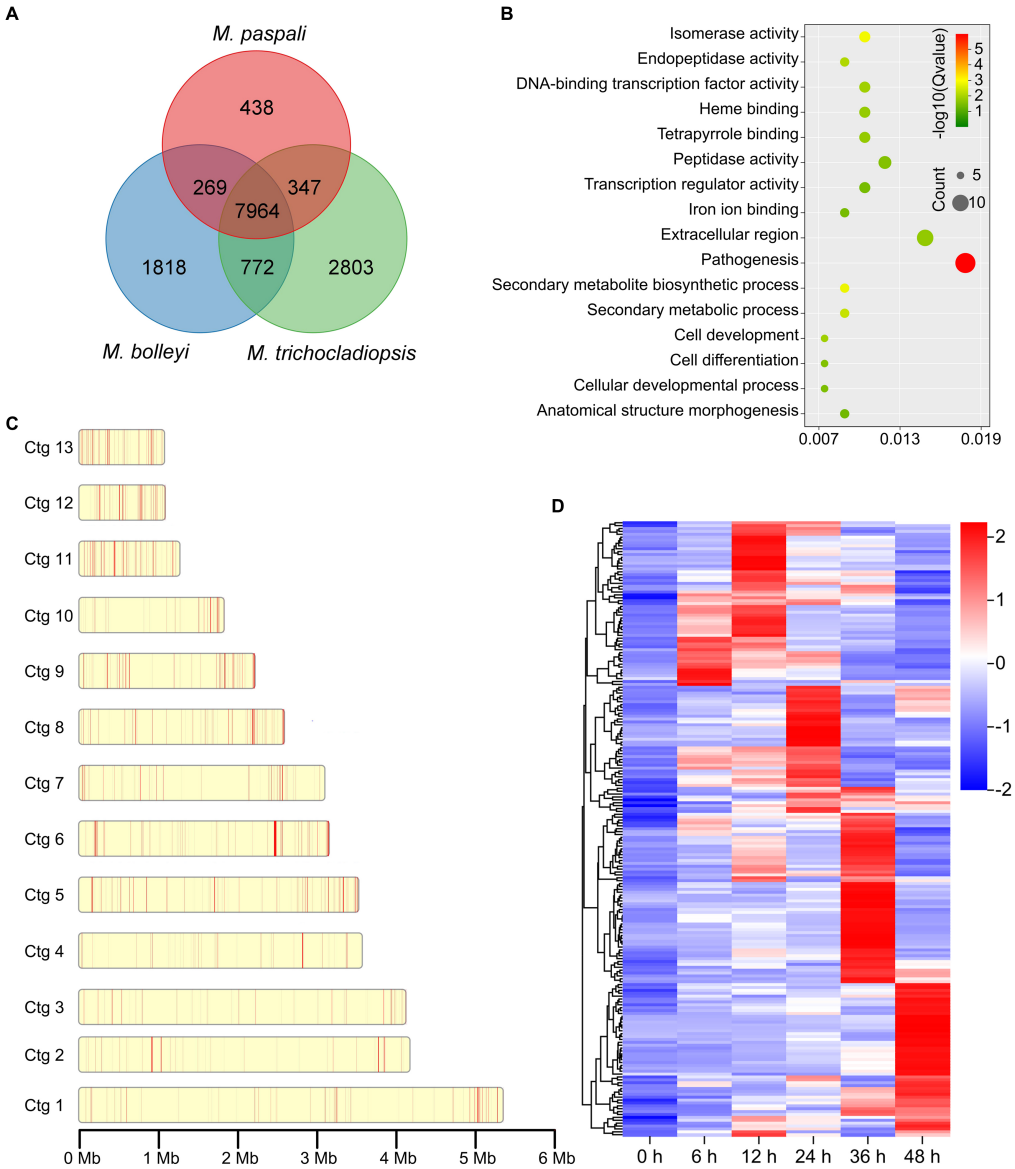
Identification and analysis of species-specific genes (SSGs) in *Microdochium paspali*. **(A)** Venn diagram for gene families clustering of the three *Microdochium* species. **(B)** Distribution of SSGs on contigs. The red color represents the position of SSGs in *M. paspali* compared with *M. bolleyi* and *M. trichocladiopsis*. **(C)** Gene Ontology (GO) enrichment analysis of SSGs in *M. paspali*. **(D)** Heat map of the normalized fragments per kilobase of transcript per million fragments mapped (FPKM) values of SSGs at different infection stages. The expression level was indicated by different colors, red indicates a high expression and blue indicates a low expression.

To further investigate the pathogenic factors in *M. paspali*, SSGs of *M. paspali* were identified and analyzed through comparison with two non-pathogenic fungal species *M. bolleyi* and *M. trichocladiopsis*. At total of 438, 1818 and 2,803 unique orthologous groups were identified in *M. paspali*, *M. bolleyi* and *M. trichocladiopsis*, respectively, which were termed as SSGs (Figure 3A). SSGs were further screened through the protein-coding genes in the *M. paspali* genome with the identity or coverage of <50% to the genes in the genomes of *M. bolleyi* and *M. trichocladiopsis* through BLAST, and 672 SSGs were finally identified in *M. paspali* (Supplementary Table S10). These SSGs were widely distributed across each contig in the *M. paspali* genome, with the most on contig 6 (Figure 3B; Supplementary Table S10).

Only 285 SSGs were annotated in the NR database and most (218 genes) had unknown functions. GO analysis showed that the functions of SSGs were classified into sixteen terms, of which twelve SSGs were significantly enriched in term of pathogenesis (Figure 3C). Eighty-six PHI homologous genes (forty-two related to pathogenicity), nine CAZymes, seventeen candidate effectors, and eighty secondary metabolite (SM) genes were identified from the SSGs in *M. paspali* (Supplementary Tables S10, S11). SSGs arranged in clusters on the genome (*n* ≥ 3) were defined as species-specific gene clusters (SSGCs), and there were 24 SSGCs identified in *M. paspali* genome (Supplementary Table S10).

**Figure 4:**
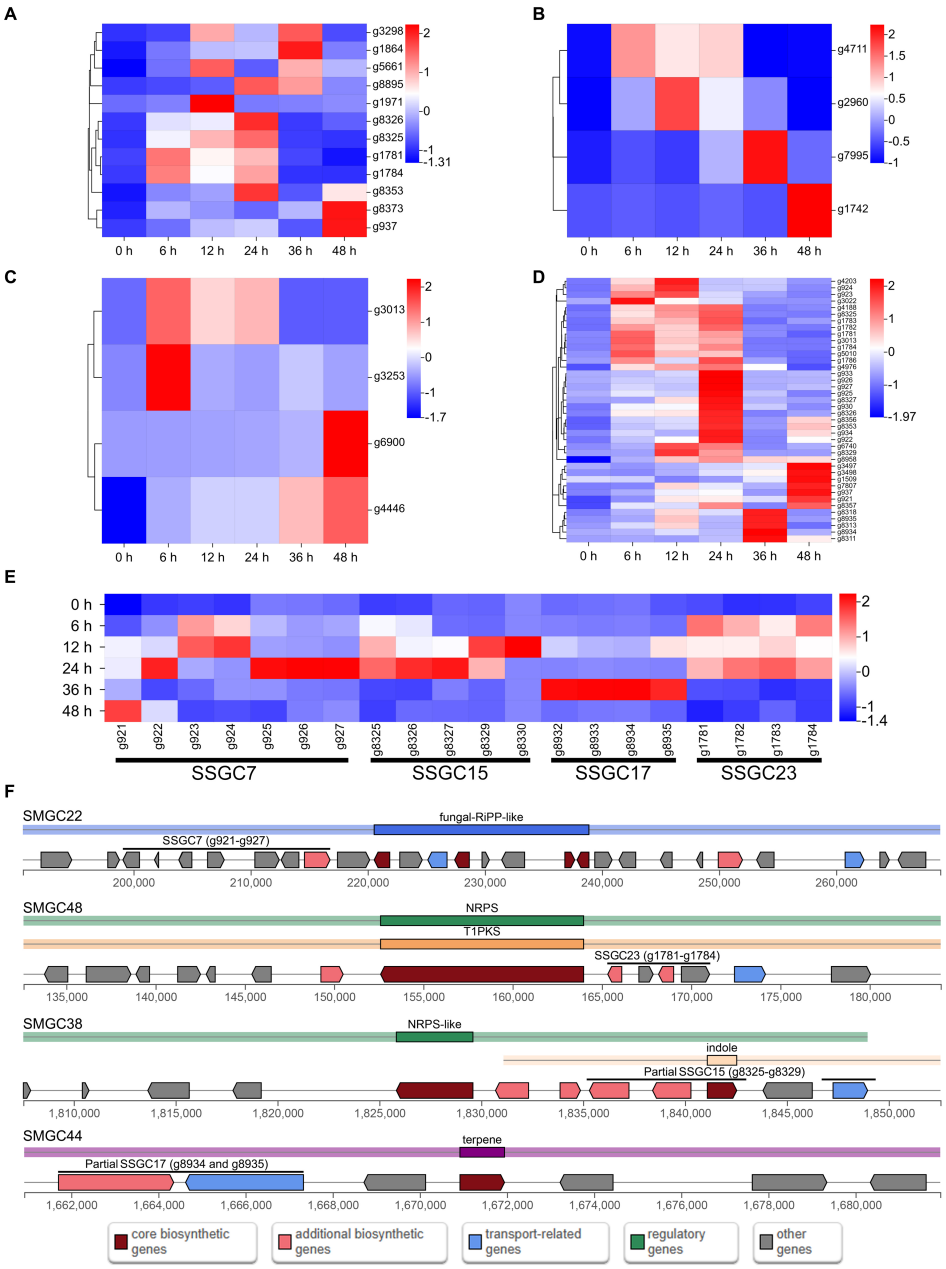
Analysis of pathogenicity-related species-specific genes (SSGs) in *Microdochium paspali*. **(A–D)** Heat map of the normalized fragments per kilobase of transcript per million fragments mapped (FPKM) values of up-regulated pathogenicity-related pathogen-host interactions (PHI) genes, carbohydrate active enzymes (CAZymes) genes, effector genes, and secondary metabolite genes in the SSGs of *M. paspali* at different infection stages. The expression level was indicated by different colors, red indicates a high expression and blue indicates a low expression. **(E)** Heat map of the four species-specific gene clusters (SSGCs) that were significantly up-regulated during the infection process. **(F)** Localization of the four SSGCs on secondary metabolite biosynthetic gene clusters (SMGCs).

The expression of most SSGs was significantly altered after infection, with two hundred and thirteen SSGs being up-regulated at least at one stage after infection (Figure 3D). Among them, there were twelve PHI pathogenicity-related genes, four CAZymes of GH family, four candidate effectors and forty SM genes (including g3022, the biosynthesis core gene of SMGC21 responsible for TeA production) (Figures 4A–D; Supplementary Table S11). Four SSGCs were specifically up-regulated (SSGC 7, SSGC 15, SSGC 17, and SSGC 23) during the infection stages (Figure 4E). Interestingly, the four SSGCs were fully or partially located on the respective SMGCs (Figure 4F), where SSGC 7 and SSGC 23 were all located on SMGC 22 and SMGC 48, while SSGC 15 and SSGC 17 were partially located on SMGC 38 and SMGC 44.

### 3.4. Comparative analyses of *Microdochium paspali* at the genomic level

**Figure 5:**
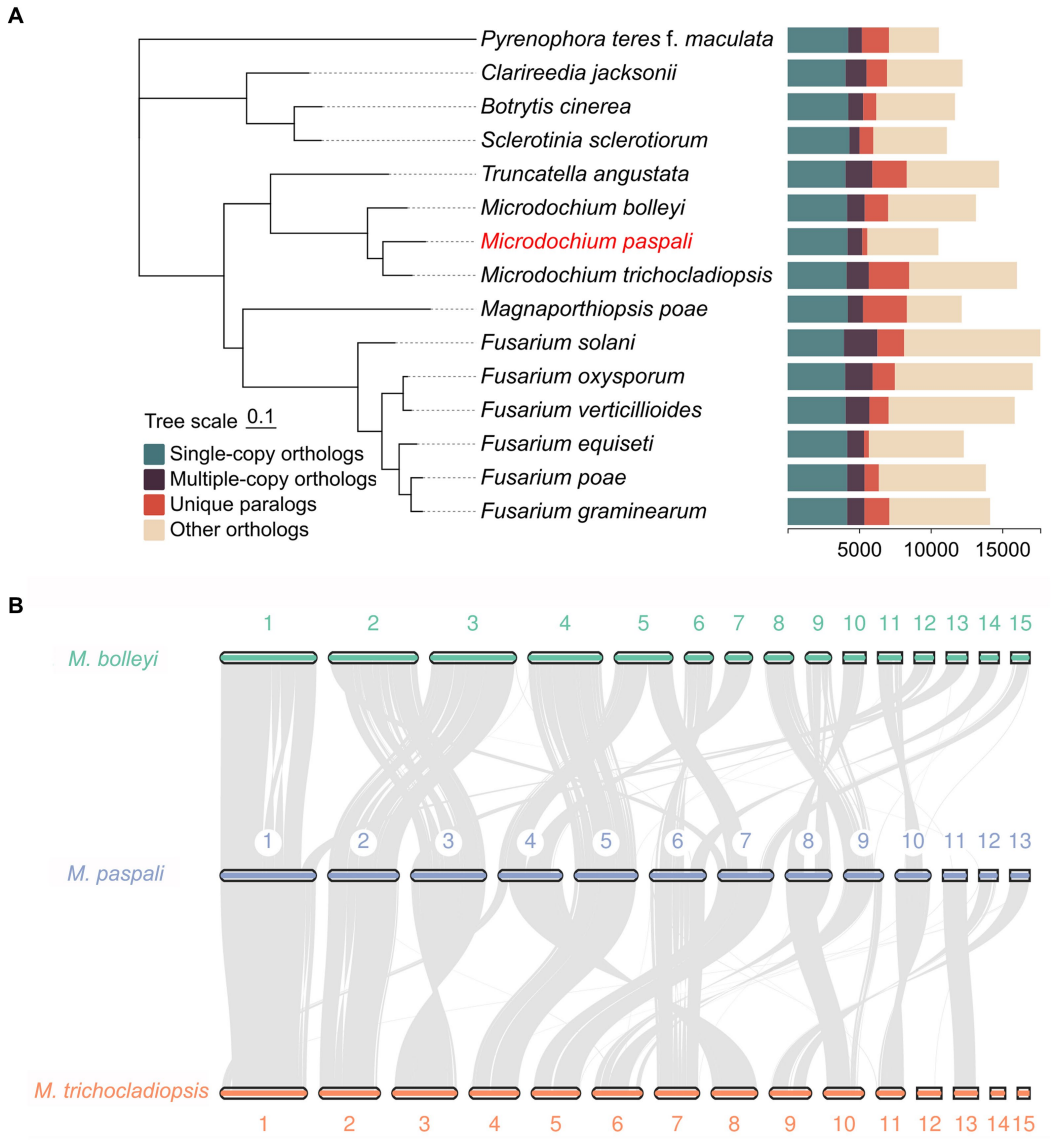
Comparative genomic analysis of *Microdochium paspali*. **(A)** Phylogenetic analysis and gene family clustering between *M. paspali* and 14 other fungi (12 plant pathogenic fungi and 2 non-pathogenic *Microdochium* species). The phylogenetic analysis was constructed based on single-copy orthologous proteins. **(B)** The genome collinearity among *M. paspali*, *M. bolleyi*, and *M. trichocladiopsis*. The thick line segments with different colors represent the contigs of each species. Each gray line connects a pair of collinearity blocks between two genomes. The first 15 long contigs of each species were selected for mapping.

The evolutionary relationship between *M. paspali* and other fungi was compared with the genomes of two non-pathogenic *Microdochium* species, *M. bolleyi* and *M. trichocladiopsis*, and 12 other representative pathogenic fungi. The phylogenetic tree was constructed according to sequence alignment of 3,024 single-copy orthologous proteins, which suggested that *M. paspali* had a close evolutionary relationship with other *Microdochium* species (Figure 5A). *M. paspali* formed a clade with *M. trichocladiopsis,* while *M. bolleyi* was a self-contained clade, indicating a closer evolutionary relationship between *M. paspali* and *M. trichocladiopsis*. In addition, *M. paspali* was also relatively close to *Truncatella angustata*, a fungal pathogen that can cause root and leaf diseases of vascular plants (Kellner et al., 2022).

Collinearity analysis revealed 8,297 collinearity gene pairs in *M. paspali* and *M. bolleyi*, and 8,575 in *M. paspali* and *M. trichocladiopsis*, further supporting the close relationship of *M. paspali* and *M. trichocladiopsis*. Compared to *M. bolleyi*, *M. paspali* had two distinct inversions on contig 1, an insertion of large fragments on contig 7, while contig 11, 12, and 13 were not collinear with *M. bolleyi*. Compared to *M. trichocladiopsis*，*M. paspali* had significant inversions on contig 3, and fusion events were identified on contig12 and contig 13, which formed contig 9 in *M. trichocladiopsis*. Furthermore, *M. paspali* showed large translocations on contig 9, as compared to both species (Figure 5B).

**Figure 6:**
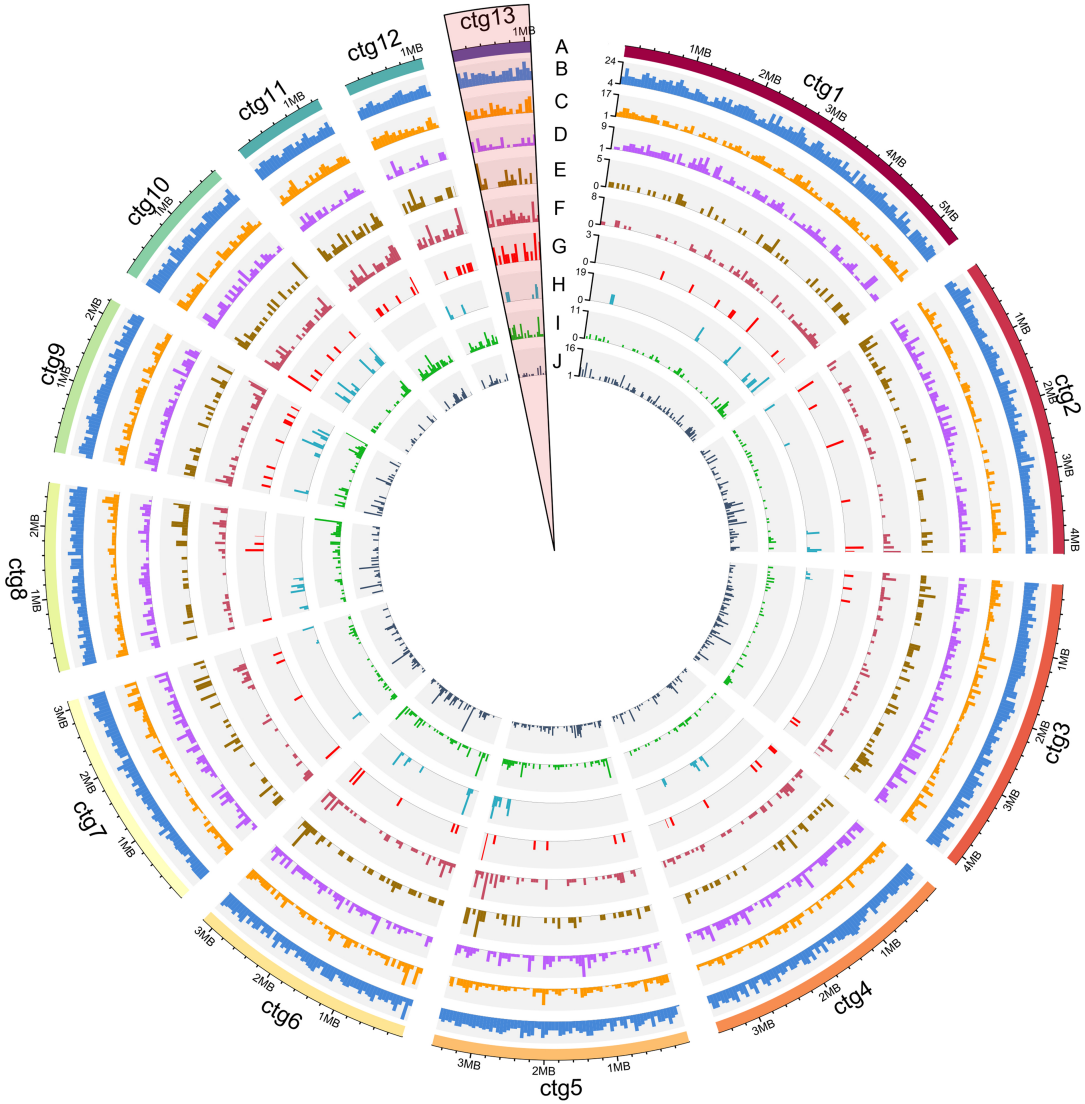
Overview of the *Microdochium paspali* genome features. The circles labeled from the outside are as follows: **(A)** the outermost layer of colored blocks is a circular representation of the 13 contigs with coding sequences, with a scale mark labeling each 200 Kb. **(B–J)** density of all genes, genes up-regulated during infection, pathogen-host interactions (PHI), carbohydrate active enzymes (CAZymes) genes, secreted protein genes, effector genes, secondary metabolite genes (SMGs), species-specific genes (SSGs), and transposable elements in each contig. All statistics are based on 50-kb non-overlapping windows.

The overview of *M. paspali* genome features revealed the enriched regions of PHI pathogenicity-related genes, CAZymes, secreted proteins (including putative effectors), SMGCs, and SSGs were mainly concentrated at the edges of contigs, and there were also more up-regulated genes during infection in these pathogenic factor-enriched regions (Figures 6C–I). Transposable elements were relatively dense in the SSGs-enriched regions, and various pathogenic factors were also more abundant (especially the SMGCs) in these regions (Figure 6J). In addition, three short contigs of *M. paspali* (contig11, contig12, and contig13) held more densely distributed SSGs and pathogenic factors than other contigs (Supplementary Table S12). There were 20 putative effectors distributed on contig13, far more than the number on other contigs (Supplementary Table S8). It is suggested that these short contigs may be related to the evolution of *M. paspali* pathogenicity.

## 4. Discussion

The genus of *Microdochium* includes some important plant pathogenic fungi which cause a variety of diseases on cereals and grasses leading to serious damage to these economically useful plants. *M. paspali* is a new pathogenic fungus causing sparse leaf patch on seashore paspalum in China (Zhang et al., 2017). However, there have been limited studies on the pathogen-host interaction process and pathogenic mechanisms of this fungus. In this study, a high-quality genome of *M. paspali* was produced containing 14 contigs and at total size of 37.32 Mb. The genome provides a valuable resource to characterize the pathogenic mechanisms of *M. paspali* and the evolutionary architectures of the genus *Microdochium*.

Comparative genomics was useful for identifying potential pathogenic factors. To investigate the potential pathogenic factors between pathogenic species *M. paspali* and non-pathogenic species *M. bolleyi* and *M. trichocladiopsis*, we conducted comparative genome analysis among the three species and identified 672 SSGs in *M. paspali*. GO analysis showed that ninety-three SSGs were annotated, and twelve SSGs were enriched in the “pathogenesis” functional category, accounting for the largest proportion. Subsequent transcriptome analysis also revealed that 31.70% SSGs (213) were up-regulated during infection, suggesting that a high number of SSGs responded to the process of *M. paspali*-seashore paspalum interaction, and might play important roles in the pathogenicity of *M. paspali*. It was worth noting that the three short contigs (contig11, 12 and 13) of *M. paspali* contained more dense SSGs and pathogenicity-related genes (up-regulated genes, CAZymes, secreted proteins, and effectors) than other contigs (Supplementary Table S12; Figure 6). Especially, there were 20 putative effectors in contig13, accounting for the largest proportion (19.04%) (Supplementary Table S8; Figure 6). The results indicate that these short contigs may be related to the pathogenicity of *M. paspali*. Further population genomics analysis is needed to characterize if high plasticity chromosome regions exist in the genome of *M. paspali* and what roles these regions play.

At initial stages of host infection, plant pathogenic fungi secrete a large number of CAZymes to degrade plant cell walls and support the processes of pathogen attachment, invasion, colonization and nutrient absorption in host plant cells (Kubicek et al., 2014). In recent years, the family of CAZymes has become an important category of pathogenic factors garnering close attention insystemic researche (Garron and Henrissat, 2019). In this study, a total of 481 CAZymes-coding genes were predicted in the *M. paspali* genome, and 198 CAZymes showed increased expression during infection. GHs accounted for the largest number in CAZymes (120 genes) and all four up-regulated SSGs encoding CAZymes belonged to GHs (g4711, g2960, g7995, and g1742). The observed result suggestes GHs may be widely involved in the interaction between *M. paspali* and seashore paspalum. Existing studies indicate GHs decomposed the cell wall during the infection of plant pathogenic fungi, helping them to invade and colonize, and also play a toxic role by inducing the immune response of the host plants (Zhao et al., 2013; Rafiei et al., 2021). Effectors are important weapons during the interaction between pathogenic fungi and host plants (Horbach et al., 2011). In this study, we found that forty putative effectors were up-regulated in at least at one time point after infection, which might be important for pathogenicity. Four of them were identified as SSGs in *M. paspali* (g3013, g3253, g4446, and g6900). However, the four SSGs have not been functionally annotated and their function needs to be further analyzed. Among the up-regulated effectors, six (g1621, g1879, g2155, g3289, g421, and g9387) were consistently up-regulated at 6–48 h after infection, which might also be the targets of interest to understand the pathogenic mechanism of *M. paspali*. In the Pfam database, g2155 is annotated as phospholipase, which can aid pathogen invasion by disrupting components of the host cell membrane, and widely exists in a variety of pathogenic fungi and bacteria (Ghannoum, 2000). The effector g3289 is annotated as trypsin, and studies reveal that the expressions of trypsin-like peptidases in fungi may represent a marker for fungal pathogenicity (Dubovenko et al., 2010). The effector g9387 was annotated as fungal hydrophobin. It is reported to be an important virulence factor in fungi and is secreted during pathogenesis. Some hydrophobin deletion mutants in *Fusarium graminearum* showed significantly reduced pathogenicity (Quarantin et al., 2019). In summary, these putative CAZymes and effectors identified by genomic and transcriptomic analyses may play important roles in the pathogenicity of *M. paspali* and will provide valuable references for subsequent studies on the pathogenic mechanisms of *M. paspali*.

During the complex processes of interaction with host plants, pathogenic fungi can produce a variety of different secondary metabolites, which affect the symptom expression and disease progression of plant hosts, and are also important virulence factors (Keller et al., 2005; Howlett, 2006). Bioinformatics predictions identified 50 different types of SMGCs in *M. paspali*, and ten showed high identities with the clusters producing known compounds. When compared with *M. bolleyi* and *M. trichocladiopsis*, it was found that *M. paspali* held a unique biosynthetic gene cluster (SMGC21). It contained thirty-one genes, and its core biosynthetic gene g3022 was a SSG of *M. paspali*, which was predicted to be responsible for the production of tenuazonic acid (TeA). TeA is a type of tetramic acidic (2,4-pyrrolidinediones) toxin that is generally considered to be a potential virulence factor (Lebrun et al., 1988; Wang et al., 2022), which can induce a chloroplast-derived oxidative burst leading to cell death and plant necrosis and has potential herbicidal activity against both monocotyledonous and dicotyledonous plants (Chen and Qiang, 2017). TeA was first reported to be isolated from *Alternaria tenuis* (Rosett et al., 1957). The plant pathogen *Phoma sorghina* (a pathogen of sorghum) and *Magnaporthe oryzae* (a pathogen of rice) were also reported to produce TeA (Umetsu et al., 1972; Steyn and Rabie, 1976). The biosynthetic gene cluster of TeA was first identified in *M. oryzae*, which contained four genes (Yun et al., 2015). Through blast analysis, we found that g3020, g3021, and g3022 in SMGC21 were 73%, 50%, and 59% similar to MGG_07802, MGG_07800, and MGG_07803 in the TeA biosynthesis gene cluster of *M. oryzae* (MIBiG accession: BGC0002158), respectively. The core biosynthetic genes of the two gene clusters, MGG_07803 and g3022, were both predicted to be the fungal non-ribosomal peptide synthetase and polyketide synthase (NRPS-PKS) hybrid enzyme. Transcriptome analysis revealed that fourteen genes (including g3020, g3021, and g3022) in SMGC21 were up-regulated after infection. Therefore, it is hypothesized that *M. paspali* has the potential to produce TeA, which may play an important role in the pathogenicity of *M. paspali*. Interestingly, we also found that four SSGCs (SSGC7, 23, 15, and 17) significantly up-regulated during infection were all partially or fully distributed on different SMGCs (SMGC22, 48, 38, and 44), suggesting that these SSGCs might be involved in the biosynthesis of certain secondary metabolic genes (SMs) in *M. paspali*. Whether the biosynthetic SMs have toxic effects on plants and what types of SMs they are will be an important direction for our future research.

## 5. Conclusion

In this study, the whole genome of *M. paspali* was sequenced for the first time. The results provide a valuable reference for genome assemblies of other related pathogenic fungi in the genus of *Microdochium*. In addition, comparative genomics and transcriptomics analyses identified large numbers of pathogenicity-related genes in *M. paspali*. These provide an important baseline for further characterization of pathogenic and evolutionary mechanisms and may assist in developing effective management strategies to control *M. paspali* on turfgrass.

## Data availability statement

The datasets presented in this study can be found in online repositories. The names of the repository/repositories and accession number(s) can be found at: https://www.ncbi.nlm.nih.gov/genbank/, JAVBIV000000000; https://www.ncbi.nlm.nih.gov/, PRJNA1007419.

## Author contributions

PJ: Data curation, Software, Writing – original draft. YK: Writing – review & editing. ZZ: Writing – review & editing. HZ: Writing – review & editing. YD: Writing – review & editing. KL: Writing – review & editing. ZY: Writing – review & editing, Conceptualization. JH: Conceptualization, Writing – review & editing, Methodology, Project administration. YZ: Resources, Formal analysis, Writing – review & editing.

## Funding

This study was supported by Jiangsu Institute of Botany Talent Fund (JIBTF202212).

## Conflict of interest

The authors declare that the research was conducted in the absence of any commercial or financial relationships that could be construed as a potential conflict of interest.

## Publisher’s note

All claims expressed in this article are solely those of the authors and do not necessarily represent those of their affiliated organizations, or those of the publisher, the editors and the reviewers. Any product that may be evaluated in this article, or claim that may be made by its manufacturer, is not guaranteed or endorsed by the publisher.

## References

[ref1] Almagro Armenteros J. J. Salvatore M. Emanuelsson O. Winther O. von Heijne G. Elofsson A.. (2019a). Detecting sequence signals in targeting peptides using deep learning. Life Sci. Alliance 2:e201900429. doi: 10.26508/lsa.201900429, PMID: 31570514 PMC6769257

[ref2] Almagro Armenteros J. J. Tsirigos K. D. Sønderby C. K. Petersen T. N. Winther O. Brunak S.. (2019b). SignalP 5.0 improves signal peptide predictions using deep neural networks. Nat. Biotechnol. 37, 420–423. doi: 10.1038/s41587-019-0036-z, PMID: 30778233

[ref3] Bao W. Kojima K. K. Kohany O. (2015). Repbase update, a database of repetitive elements in eukaryotic genomes. Mob. DNA 6:11. doi: 10.1186/s13100-015-0041-9 26045719 PMC4455052

[ref4] Blin K. Shaw S. Kloosterman A. M. Charlop-Powers Z. van Wezel G. P. Medema M. H.. (2021). antiSMASH 6.0: improving cluster detection and comparison capabilities. Nucleic Acids Res. 49, W29–W35. doi: 10.1093/nar/gkab335, PMID: 33978755 PMC8262755

[ref5] Brůna T. Hoff K. J. Lomsadze A. Stanke M. Borodovsky M. (2021). BRAKER2: automatic eukaryotic genome annotation with GeneMark-EP+ and AUGUSTUS supported by a protein database. NAR Genom. Bioinf. 3:lqaa108. doi: 10.1093/nargab/lqaa108 PMC7787252 33575650

[ref6] Buchfink B. Xie C. Huson D. (2015). Fast and sensitive protein alignment using DIAMOND. Nat. Methods 12, 59–60. doi: 10.1038/nmeth.3176, PMID: 25402007

[ref7] Camacho C. Coulouris G. Avagyan V. Ma N. Papadopoulos J. Bealer K.. (2009). BLAST+: architecture and applications. BMC Bioinformatics 10:421. doi: 10.1186/1471-2105-10-421 20003500 PMC2803857

[ref8] Chen C. Chen H. Zhang Y. Thomas H. R. Frank M. H. He Y.. (2020). TBtools: an integrative toolkit developed for interactive analyses of big biological data. Mol. Plant 13, 1194–1202. doi: 10.1016/j.molp.2020.06.009, PMID: 32585190

[ref9] Chen Y. Nie F. Xie S.-Q. Zheng Y.-F. Dai Q. Bray T.. (2021). Efficient assembly of nanopore reads via highly accurate and intact error correction. Nat. Commun. 12:60. doi: 10.1038/s41467-020-20236-7 33397900 PMC7782737

[ref10] Chen S. Qiang S. (2017). Recent advances in tenuazonic acid as a potential herbicide. Pestic. Biochem. Physiol. 143, 252–257. doi: 10.1016/j.pestbp.2017.01.003, PMID: 29183600

[ref11] David A. S. Haridas S. LaButti K. Lim J. Lipzen A. Wang M.. (2016). Draft genome sequence of Microdochium bolleyi, a dark septate fungal endophyte of beach grass. Genome Announc. 4, e00270–e00216. doi: 10.1128/genomeA.00270-16 27125481 PMC4850852

[ref12] Dubovenko A. G. Dunaevsky Y. E. Belozersky M. A. Oppert B. Lord J. C. Elpidina E. N. (2010). Trypsin-like proteins of the fungi as possible markers of pathogenicity. Fungal Biol. 114, 151–159. doi: 10.1016/j.funbio.2009.11.004, PMID: 20960971

[ref13] Edgar R. C. (2004). MUSCLE: multiple sequence alignment with high accuracy and high throughput. Nucleic Acids Res. 32, 1792–1797. doi: 10.1093/nar/gkh340, PMID: 15034147 PMC390337

[ref14] Emms D. M. Kelly S. (2019). OrthoFinder: phylogenetic orthology inference for comparative genomics. Genome Biol. 20:238. doi: 10.1186/s13059-019-1832-y 31727128 PMC6857279

[ref15] Flynn J. M. Hubley R. Goubert C. Rosen J. Clark A. G. Feschotte C.. (2020). RepeatModeler2 for automated genomic discovery of transposable element families. Proc. Natl. Acad. Sci. U. S. A. 117, 9451–9457. doi: 10.1073/pnas.1921046117, PMID: 32300014 PMC7196820

[ref16] Garron M.-L. Henrissat B. (2019). The continuing expansion of CAZymes and their families. Curr. Opin. Chem. Biol. 53, 82–87. doi: 10.1016/j.cbpa.2019.08.004, PMID: 31550558

[ref17] Ghannoum M. A. (2000). Potential role of phospholipases in virulence and fungal pathogenesis. Clin. Microbiol. Rev. 13, 122–143. doi: 10.1128/CMR.13.1.122, PMID: 10627494 PMC88936

[ref18] Horbach R. Navarro-Quesada A. R. Knogge W. Deising H. B. (2011). When and how to kill a plant cell: infection strategies of plant pathogenic fungi. J. Plant Physiol. 168, 51–62. doi: 10.1016/j.jplph.2010.06.014, PMID: 20674079

[ref19] Howlett B. J. (2006). Secondary metabolite toxins and nutrition of plant pathogenic fungi. Curr. Opin. Plant Biol. 9, 371–375. doi: 10.1016/j.pbi.2006.05.004, PMID: 16713733

[ref20] Huang S. Xia J. Zhang X. Sun W. Li Z. (2020). Two new species of *Microdochium* from *Indocalamus longiauritus* in South-Western China. MycoKeys 72, 93–108. doi: 10.3897/mycokeys.72.55445, PMID: 32982557 PMC7498474

[ref21] Jadubansa P. Lethbridge G. Bushell M. E. (1994). Physiology of production of viable biomass and spore inoculum for the biocontrol agent *Idriella (Microdochium) bolleyi*. Enzym. Microb. Technol. 16, 24–28. doi: 10.1016/0141-0229(94)90105-8

[ref22] Jewell L. E. Hsiang T. (2013). Multigene differences between *Microdochium nivale* and *Microdochium majus*. Botany 91, 99–106. doi: 10.1139/cjb-2012-0178

[ref23] Johnson L. S. Eddy S. R. Portugaly E. (2010). Hidden Markov model speed heuristic and iterative HMM search procedure. BMC Bioinformatics 11:431. doi: 10.1186/1471-2105-11-431 20718988 PMC2931519

[ref24] Jones D. A. B. Bertazzoni S. Turo C. J. Syme R. A. Hane J. K. (2018). Bioinformatic prediction of plant–pathogenicity effector proteins of fungi. Curr. Opin. Microbiol. 46, 43–49. doi: 10.1016/j.mib.2018.01.017, PMID: 29462764

[ref25] Jones P. Binns D. Chang H.-Y. Fraser M. Li W. McAnulla C.. (2014). InterProScan 5: genome-scale protein function classification. Bioinformatics 30, 1236–1240. doi: 10.1093/bioinformatics/btu031, PMID: 24451626 PMC3998142

[ref26] Kammerer S. J. Burpee L. L. Harmon P. F. (2011). Identification of a new *Waitea circinata* variety causing basal leaf blight of seashore paspalum. Plant Dis. 95, 515–522. doi: 10.1094/PDIS-03-10-0204, PMID: 30731941

[ref27] Karimi I. Y. M. Kurup S. S. Salem M. A. M. A. Cheruth A. J. Purayil F. T. Subramaniam S.. (2018). Evaluation of Bermuda and paspalum grass types for urban landscapes under saline water irrigation. J. Plant Nutr. 41, 888–902. doi: 10.1080/01904167.2018.1431669

[ref28] Keller N. P. Turner G. Bennett J. W. (2005). Fungal secondary metabolism — from biochemistry to genomics. Nat. Rev. Microbiol. 3, 937–947. doi: 10.1038/nrmicro1286, PMID: 16322742

[ref29] Kellner H. Friedrich S. Schmidtke K. U. Ullrich R. Kiebist J. Zänder D.. (2022). Draft genome sequence of *Truncatella angustata* (anamorph) S358. Microbiol. Resour. Announc. 11, e00052–e00022. doi: 10.1128/mra.00052-22 35658563 PMC9302084

[ref30] Kim D. Langmead B. Salzberg S. L. (2015). HISAT: a fast spliced aligner with low memory requirements. Nat. Methods 12, 357–360. doi: 10.1038/nmeth.3317, PMID: 25751142 PMC4655817

[ref31] Krogh A. Larsson B. von Heijne G. Sonnhammer E. L. (2001). Predicting transmembrane protein topology with a hidden Markov model: application to complete genomes. J. Mol. Biol. 305, 567–580. doi: 10.1006/jmbi.2000.4315, PMID: 11152613

[ref32] Kubicek C. P. Starr T. L. Glass N. L. (2014). Plant cell wall-degrading enzymes and their secretion in plant-pathogenic fungi. Annu. Rev. Phytopathol. 52, 427–451. doi: 10.1146/annurev-phyto-102313-045831, PMID: 25001456

[ref33] Lebrun M. H. Nicolas L. Boutar M. Gaudemer F. Ranomenjanahary S. Gaudemer A. (1988). Relationships between the structure and the phytotoxicity of the fungal toxin tenuazonic acid. Phytochemistry 27, 77–84. doi: 10.1016/0031-9422(88)80594-6, PMID: 36100332

[ref34] Li R. Li Y. Fang X. Yang H. Wang J. Kristiansen K.. (2009). SNP detection for massively parallel whole-genome resequencing. Genome Res. 19, 1124–1132. doi: 10.1101/gr.088013.108, PMID: 19420381 PMC2694485

[ref35] Liang J. Li G. Zhao M. Cai L. (2019). A new leaf blight disease of turfgrasses caused by *Microdochium poae*, sp. nov. Mycologia 111, 265–273. doi: 10.1080/00275514.2019.1569417, PMID: 30856060

[ref36] Lonard R. I. Judd F. W. Stalter R. (2015). Biological flora of coastal dunes and wetlands: *Paspalum vaginatum* Sw. J. Coast. Res. 31, 213–223. doi: 10.2112/JCOASTRES-D-14-00022.1

[ref37] Love M. I. Huber W. Anders S. (2014). Moderated estimation of fold change and dispersion for RNA-seq data with DESeq2. Genome Biol. 15:550. doi: 10.1186/s13059-014-0550-8 25516281 PMC4302049

[ref38] Mesny F. Miyauchi S. Thiergart T. Pickel B. Atanasova L. Karlsson M.. (2021). Genetic determinants of endophytism in the *Arabidopsis* root mycobiome. Nat. Commun. 12:7227. doi: 10.1038/s41467-021-27479-y 34893598 PMC8664821

[ref39] Mistry J. Chuguransky S. Williams L. Qureshi M. Salazar G. A. Sonnhammer E. L. L.. (2021). Pfam: the protein families database in 2021. Nucleic Acids Res. 49, D412–D419. doi: 10.1093/nar/gkaa913, PMID: 33125078 PMC7779014

[ref40] Müller E. Samuels G. J. (1984). *Monographella maydis* sp. nov. and its connection to the tar-spot disease of *Zea mays*. Nova Hedwigia 40, 113–121.

[ref41] Murray M. G. Thompson W. F. (1980). Rapid isolation of high molecular weight plant DNA. Nucleic Acids Res. 8, 4321–4325. doi: 10.1093/nar/8.19.4321, PMID: 7433111 PMC324241

[ref42] Nawrocki E. P. Eddy S. R. (2013). Infernal 1.1: 100-fold faster RNA homology searches. Bioinformatics 29, 2933–2935. doi: 10.1093/bioinformatics/btt509, PMID: 24008419 PMC3810854

[ref43] Parkinson V. O. (1980). Cultural characteristics of the rice leaf scald fungus, *Rhynchosporium oryzae*. Trans. Br. Mycol. Soc. 74, 509–514. doi: 10.1016/S0007-1536(80)80050-7

[ref44] Pertea M. Pertea G. Antonescu C. Chang T.-C. Mendell J. T. Salzberg S. L. (2015). StringTie enables improved reconstruction of a transcriptome from RNA-seq reads. Nat. Biotechnol. 33, 290–295. doi: 10.1038/nbt.3122, PMID: 25690850 PMC4643835

[ref45] Pierleoni A. Martelli P. L. Casadio R. (2008). PredGPI: a GPI-anchor predictor. BMC Bioinformatics 9:392. doi: 10.1186/1471-2105-9-392 18811934 PMC2571997

[ref46] Pompeiano A. Di Patrizio E. Volterrani M. Scartazza A. Guglielminetti L. (2016). Growth responses and physiological traits of seashore paspalum subjected to short-term salinity stress and recovery. Agric. Water Manag. 163, 57–65. doi: 10.1016/j.agwat.2015.09.004

[ref47] Quarantin A. Hadeler B. Kröger C. Schäfer W. Favaron F. Sella L.. (2019). Different Hydrophobins of *fusarium graminearum* are involved in hyphal growth, attachment, water-air interface penetration and plant infection. Front. Microbiol. 10:751. doi: 10.3389/fmicb.2019.00751 31031728 PMC6474331

[ref48] Rafiei V. Velez H. Tzelepis G. (2021). The role of glycoside hydrolases in phytopathogenic fungi and oomycetes virulence. Int. J. Mol. Sci. 22:9359. doi: 10.3390/ijms22179359, PMID: 34502268 PMC8431085

[ref49] Roach M. J. Schmidt S. A. Borneman A. R. (2018). Purge Haplotigs: allelic contig reassignment for third-gen diploid genome assemblies. BMC Bioinformatics 19:460. doi: 10.1186/s12859-018-2485-7 30497373 PMC6267036

[ref50] Rosett T. Sankhala R. H. Stickings C. E. Taylor M. E. U. Thomas R. (1957). Biochemistry of microorganisms. CIII. Metabolites of *Alternaria tenuis* auct.: culture filtrate products. Biochem. J. 67, 390–400. doi: 10.1042/bj0670390, PMID: 13479395 PMC1200169

[ref51] Shen Y. Liu N. Li C. Wang X. Xu X. Chen W.. (2017). The early response during the interaction of fungal phytopathogen and host plant. Open Biol. 7:170057. doi: 10.1098/rsob.170057, PMID: 28469008 PMC5451545

[ref52] Simão F. A. Waterhouse R. M. Ioannidis P. Kriventseva E. V. Zdobnov E. M. (2015). BUSCO: assessing genome assembly and annotation completeness with single-copy orthologs. Bioinformatics 31, 3210–3212. doi: 10.1093/bioinformatics/btv351, PMID: 26059717

[ref53] Sperschneider J. Dodds P. N. (2022). EffectorP 3.0: prediction of apoplastic and cytoplasmic effectors in fungi and oomycetes. Mol. Plant-Microbe Interact. 35, 146–156. doi: 10.1094/MPMI-08-21-0201-R, PMID: 34698534

[ref54] Stamatakis A. (2014). RAxML version 8: a tool for phylogenetic analysis and post-analysis of large phylogenies. Bioinformatics 30, 1312–1313. doi: 10.1093/bioinformatics/btu033, PMID: 24451623 PMC3998144

[ref55] Steyn P. S. Rabie C. (1976). Characterization of magnesium and calcium tenuazonate from Phoma sorghina. J. Phytochem. 15, 1977–1979. doi: 10.1016/S0031-9422(00)88860-3

[ref56] Tempel S. (2012). Using and understanding repeat masker. Methods Mol. Biol. 859, 29–51. doi: 10.1007/978-1-61779-603-6_2 22367864

[ref57] Umetsu N. Kaji J. Tamari K. (1972). Investigation on the toxin production by several blast fungus strains and isolation of tenuazonic acid as a novel toxin. Agric. Biol. Chem. 36, 859–866. doi: 10.1080/00021369.1972.10860315

[ref58] Urban M. Cuzick A. Seager J. Wood V. Rutherford K. Venkatesh S. Y.. (2021). PHI-base in 2022: a multi-species phenotype database for pathogen–host interactions. Nucleic Acids Res. 50, D837–D847. doi: 10.1093/nar/gkab1037 PMC8728202 34788826

[ref59] Vaser R. Sović I. Nagarajan N. Šikić M. (2017). Fast and accurate de novo genome assembly from long uncorrected reads. Genome Res. 27, 737–746. doi: 10.1101/gr.214270.116, PMID: 28100585 PMC5411768

[ref60] Vurture G. W. Sedlazeck F. J. Nattestad M. Underwood C. J. Fang H. Gurtowski J.. (2017). GenomeScope: fast reference-free genome profiling from short reads. Bioinformatics 33, 2202–2204. doi: 10.1093/bioinformatics/btx153, PMID: 28369201 PMC5870704

[ref61] Walker B. J. Abeel T. Shea T. Priest M. Abouelliel A. Sakthikumar S.. (2014). Pilon: an integrated tool for comprehensive microbial variant detection and genome assembly improvement. PLoS One 9:e112963. doi: 10.1371/journal.pone.0112963, PMID: 25409509 PMC4237348

[ref62] Wang S. J. (2015). First report of take-all root rot caused by *Gaeumannomyces graminis* var. *graminis* on *Paspalum vaginatum* in China. Plant Dis. 99:1858. doi: 10.1094/PDIS-01-15-0083-PDN

[ref63] Wang H. Guo Y. Luo Z. Gao L. Li R. Zhang Y.. (2022). Recent advances in *Alternaria* phytotoxins: a review of their occurrence, structure, bioactivity, and biosynthesis. J. Fungi 8:168. doi: 10.3390/jof8020168, PMID: 35205922 PMC8878860

[ref64] Wang Y. Jia L. Tian G. Dong Y. Zhang X. Zhou Z.. (2023). shinyCircos-V2.0: leveraging the creation of Circos plot with enhanced usability and advanced features. iMeta. 2:e109. doi: 10.1002/imt2.109 PMC10989951 38868422

[ref65] Wang Y. Tang H. DeBarry J. D. Tan X. Li J. Wang X.. (2012). MCScanX: a toolkit for detection and evolutionary analysis of gene synteny and collinearity. Nucleic Acids Res. 40:e49. doi: 10.1093/nar/gkr1293, PMID: 22217600 PMC3326336

[ref66] Yun C. S. Motoyama T. Osada H. (2015). Biosynthesis of the mycotoxin tenuazonic acid by a fungal NRPS-PKS hybrid enzyme. Nat. Commun. 6:8758. doi: 10.1038/ncomms9758 26503170 PMC4640141

[ref67] Zhang H. Dong Y. Zhou Y. Hu J. Lamour K. Yang Z. (2022). *Clarireedia hainanense*: a new species is associated with dollar spot of turfgrass in Hainan, China. Plant Dis. 106, 996–1002. doi: 10.1094/PDIS-08-21-1853-RE, PMID: 34698519

[ref68] Zhang W. Nan Z. Tian P. Hu M. Gao Z. Li M.. (2017). *Microdochium paspali*, a new species causing seashore paspalum disease in southern China. Mycologia 107, 80–89. doi: 10.3852/14-119 25261493

[ref69] Zhang H. Yohe T. Huang L. Entwistle S. Wu P. Yang Z.. (2018). dbCAN2: a meta server for automated carbohydrate-active enzyme annotation. Nucleic Acids Res. 46, W95–W101. doi: 10.1093/nar/gky418, PMID: 29771380 PMC6031026

[ref70] Zhao Z. Liu H. Wang C. Xu J.-R. (2013). Comparative analysis of fungal genomes reveals different plant cell wall degrading capacity in fungi. BMC Genomics 14:274. doi: 10.1186/1471-2164-14-274 23617724 PMC3652786

