# Supplementary material for: Comparative genomics and transcriptome analysis reveals potential pathogenic mechanisms of Microdochium paspali on seashore paspalum
Source: Front Microbiol. 2023 Sep 15;14:1259241. doi: 10.3389/fmicb.2023.1259241 (PMC10546424; doi:10.3389/fmicb.2023.1259241)
Supplement: Supplementary file 1 [file Image_1.pdf]

Supplementary Material - Figures S1 – S8

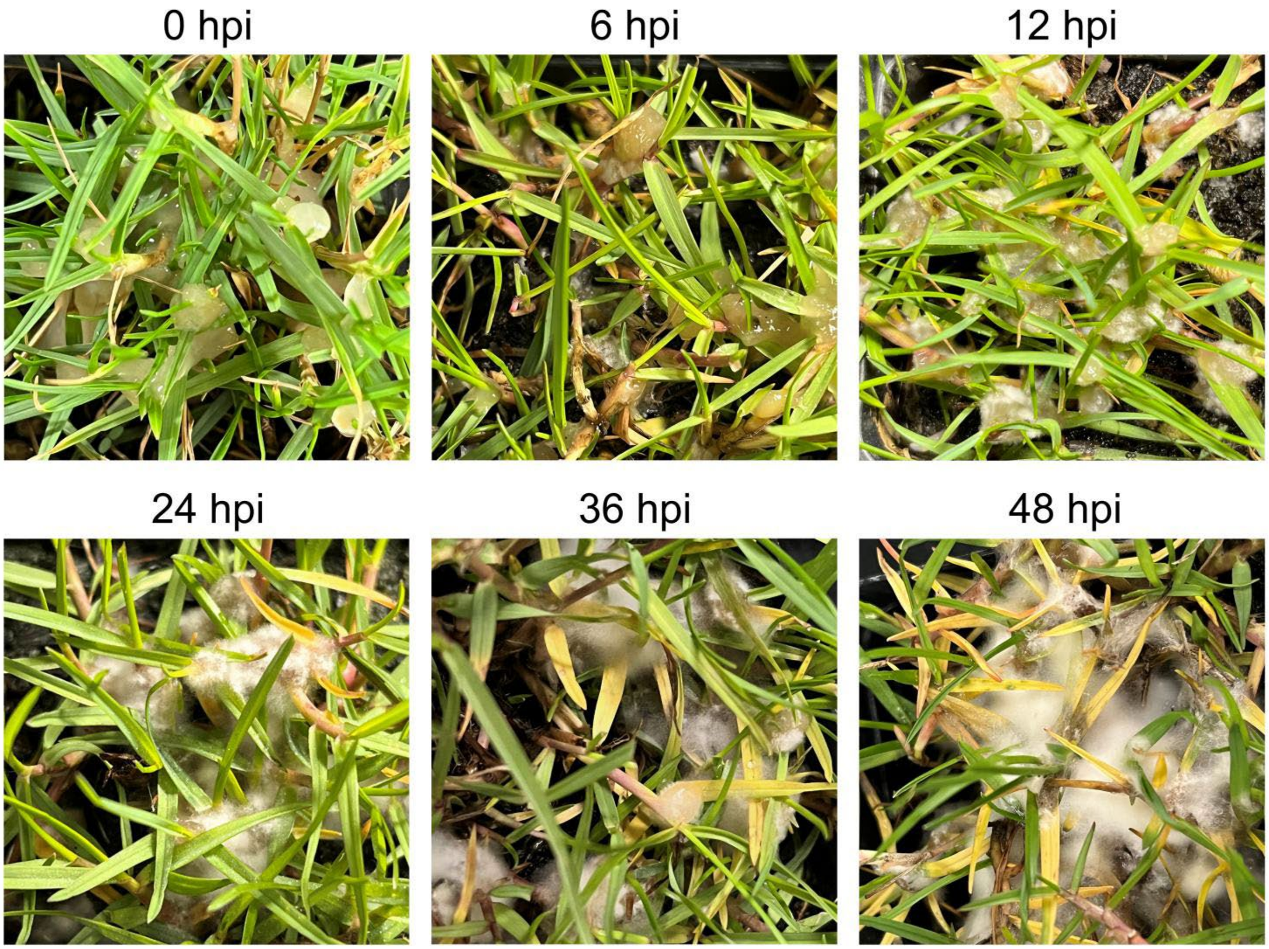

Figure S1. Symptoms of seashore paspalum at 6h, 12h, 24h, 36h, and 48h after *Microdochium paspali* infection.

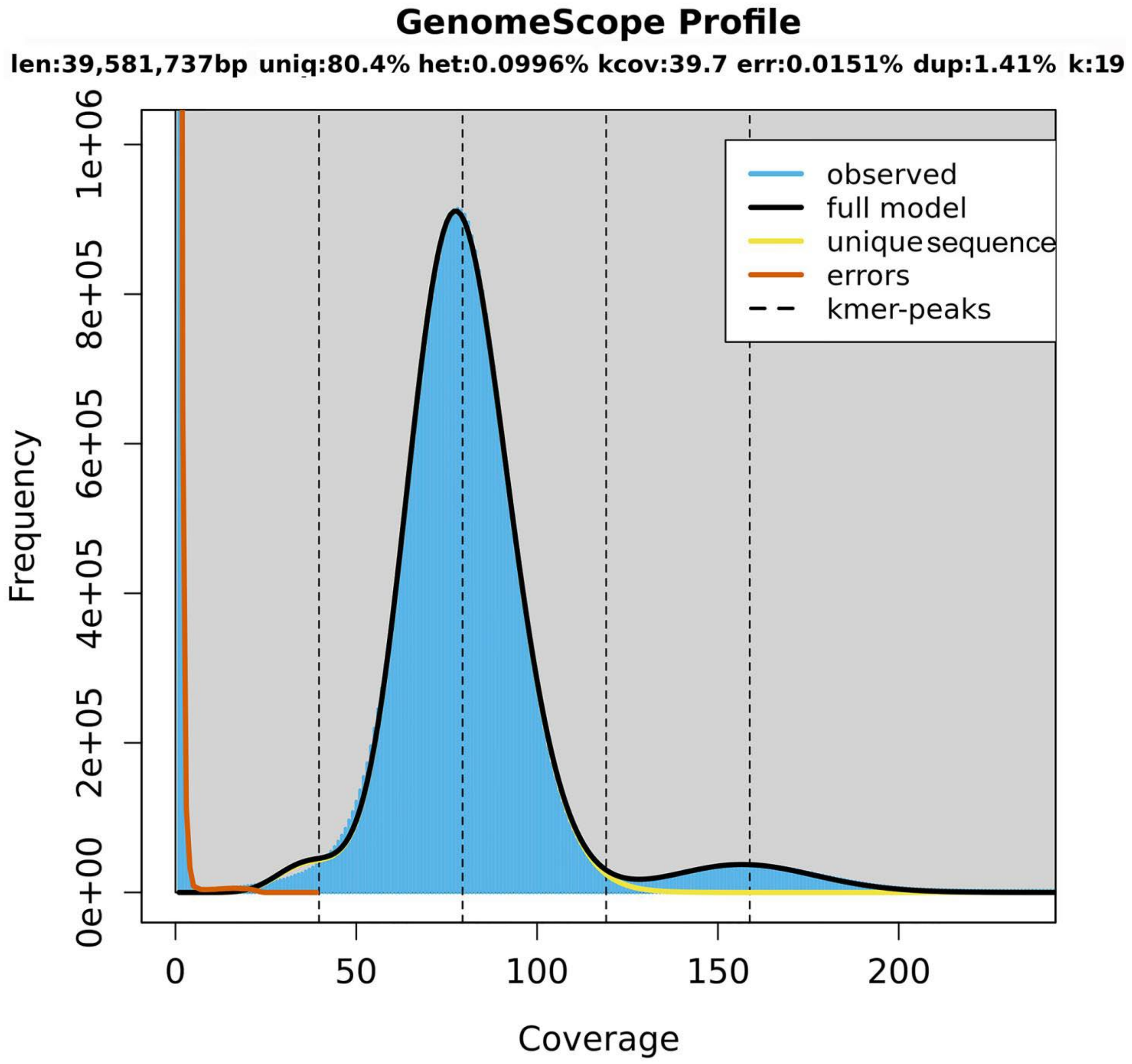

Figure S2. K-mer analysis of the *Microdochium paspali* genome using Jellyfish software.

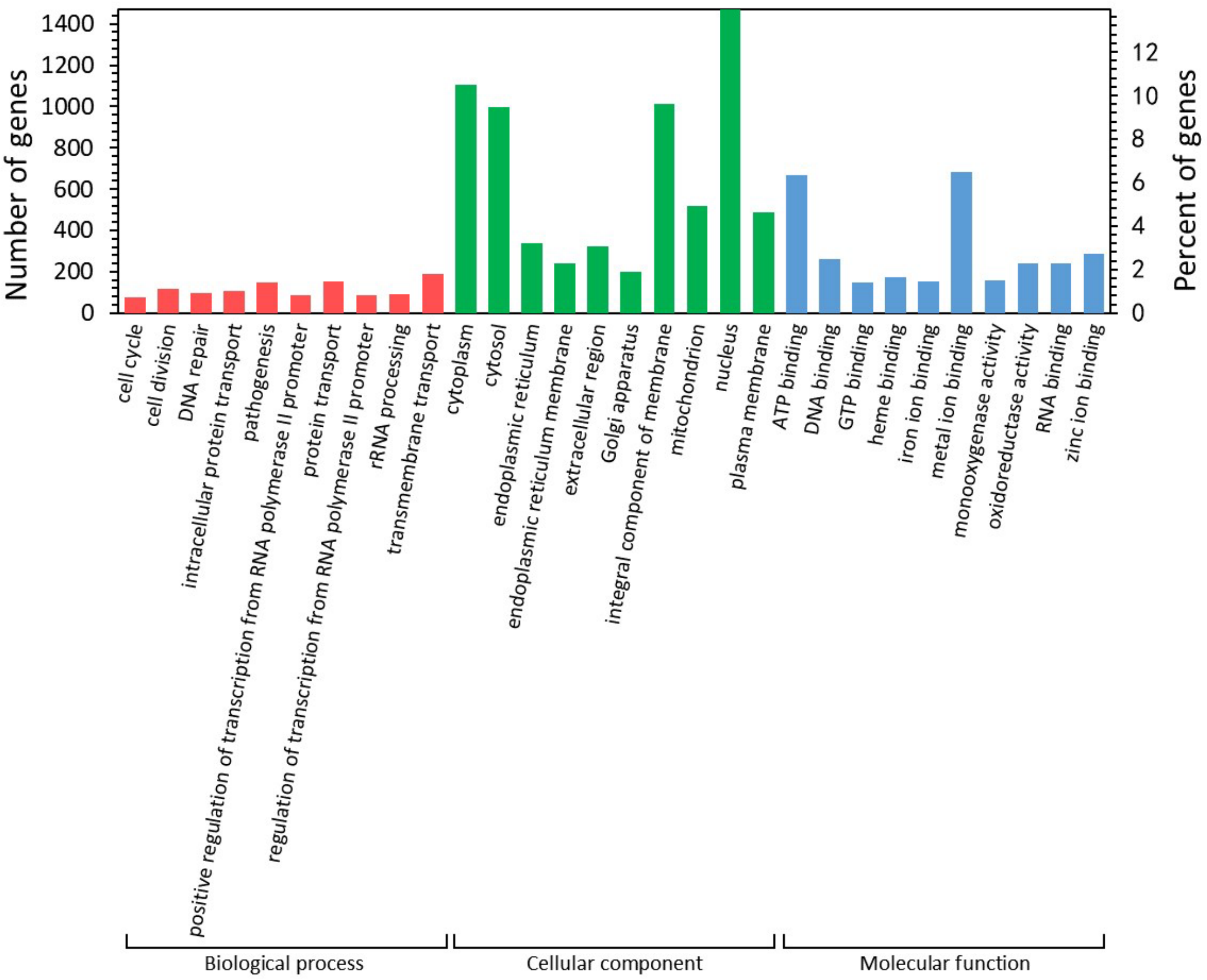

Figure S3. GO functional analysis of *Microdochium paspali*.

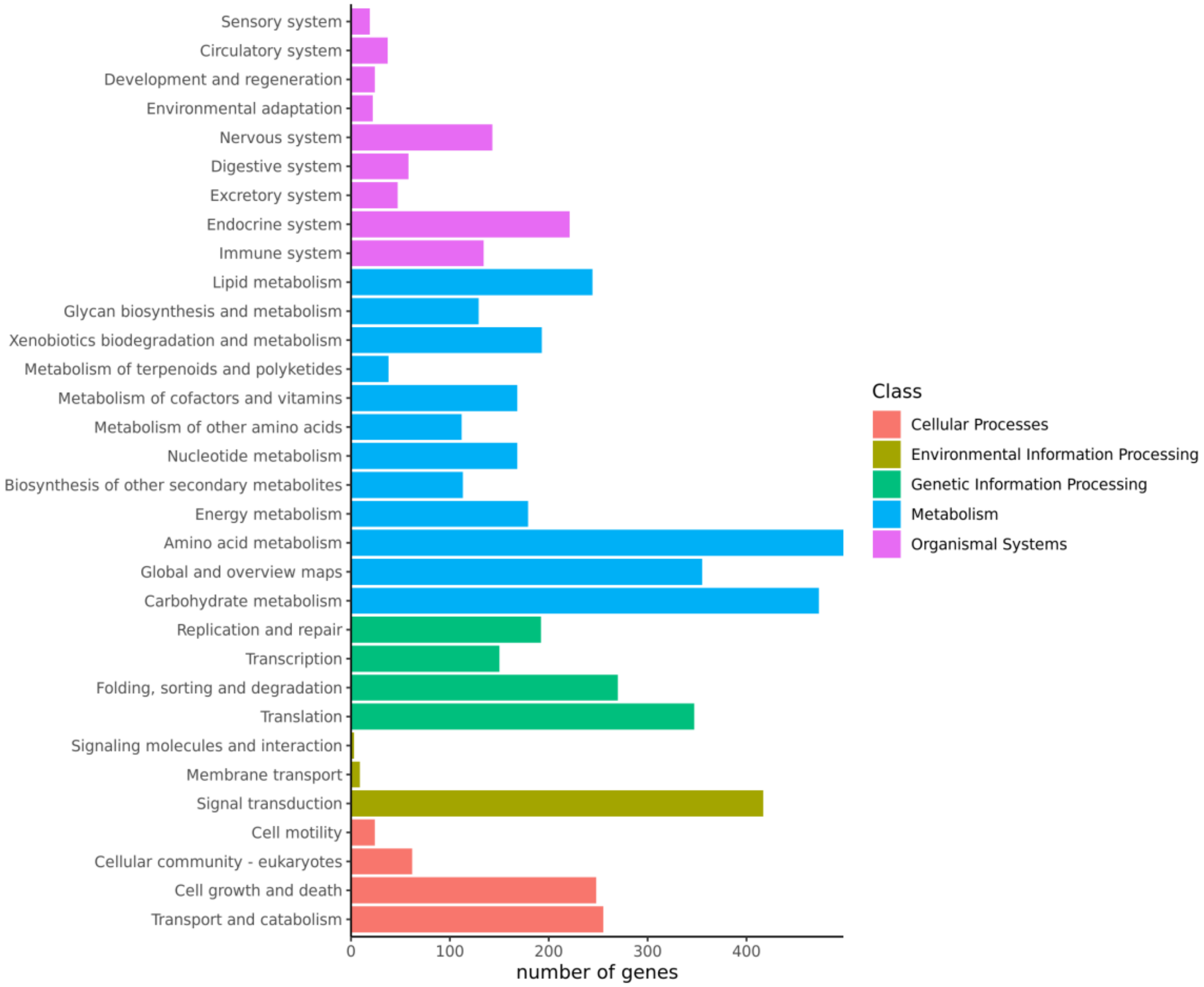

Figure S4. KEGG functional analysis of *Microdochium paspali*.

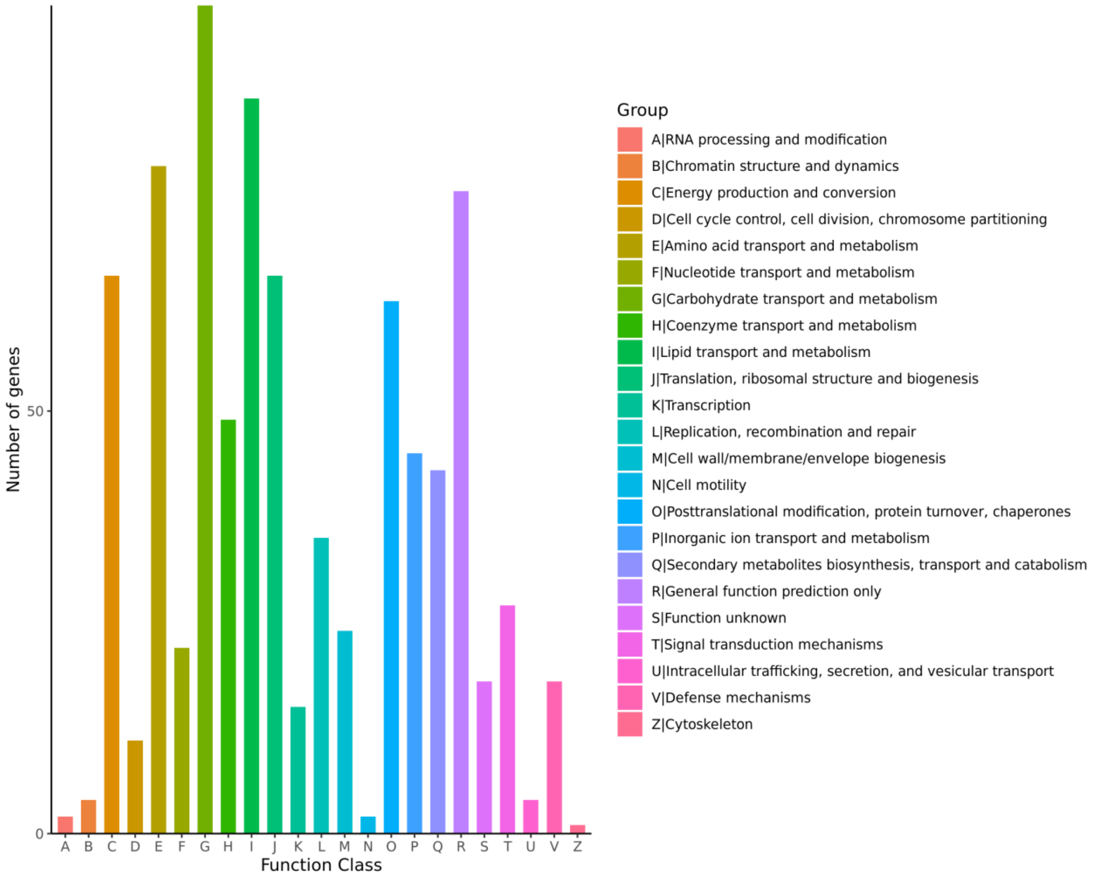

Figure S5. COG functional analysis of *Microdochium paspali*.

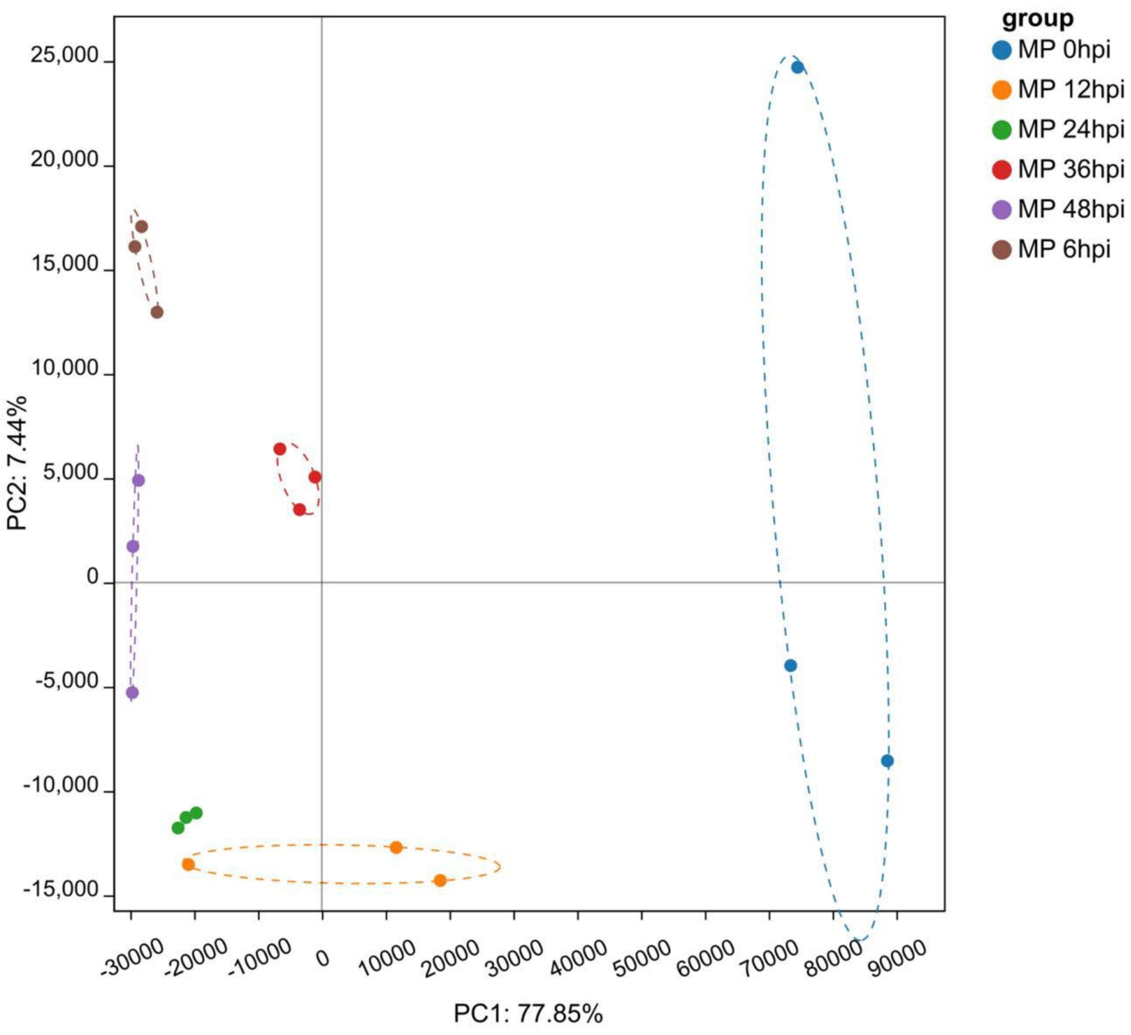

Figure S6. Principal component analysis based on FPKM values of different samples at six time points after infection.

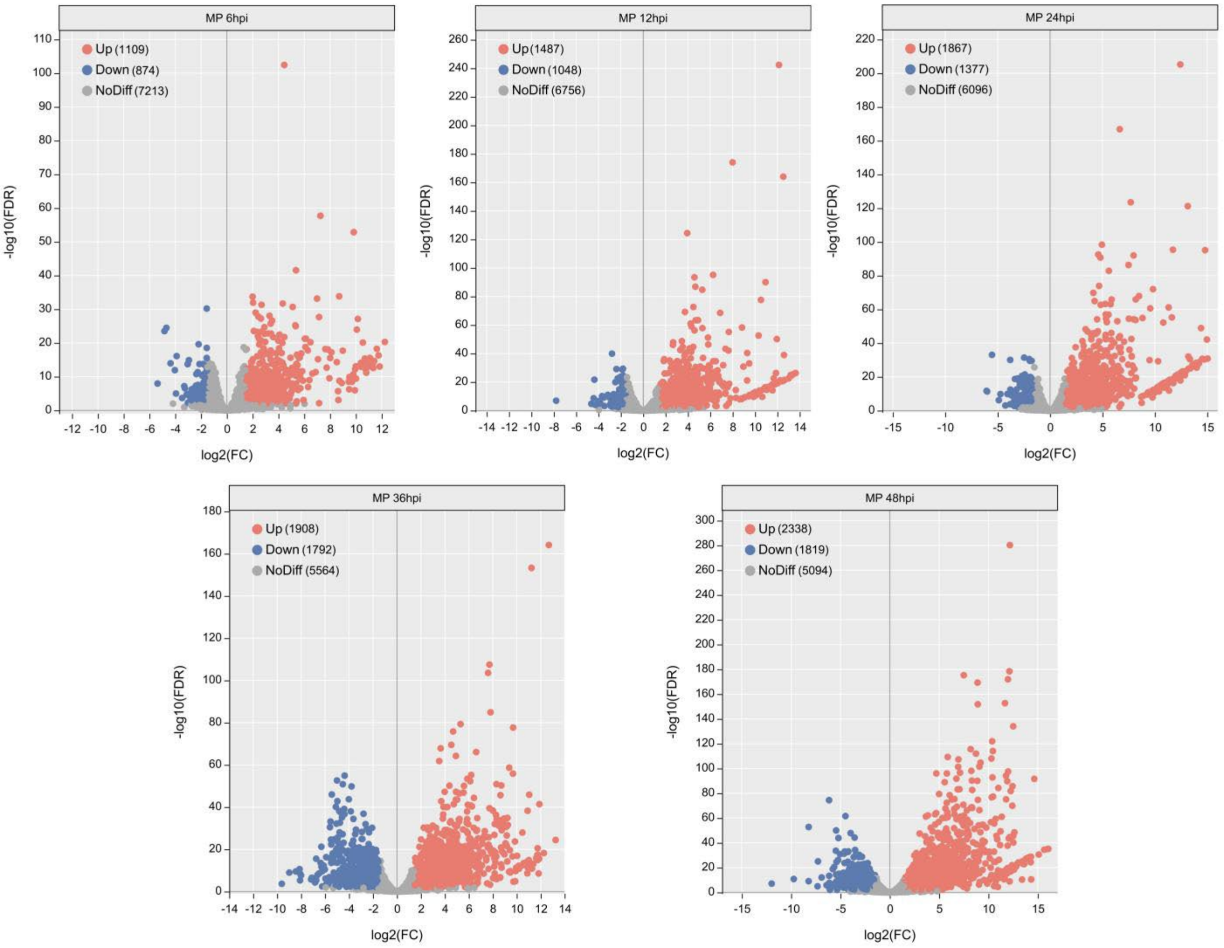

Figure S7. Volcano plot of differentially expressed genes at 6h, 12h, 24h, 36h, and 48h after *Microdochium paspali* infection on seashore paspalum.

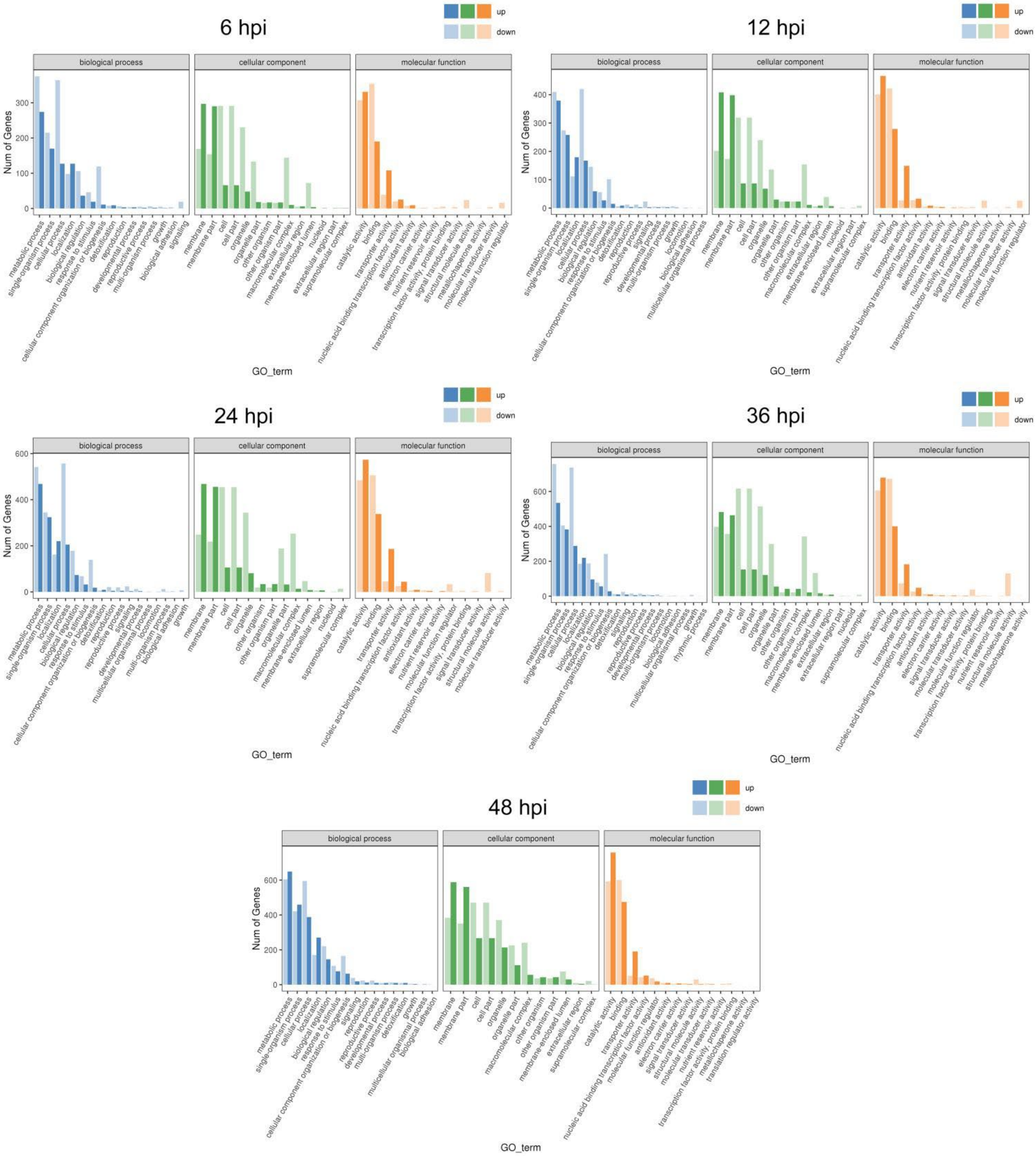

Figure S8. GO enrichment analysis of differentially expressed genes (DEGs) during *Microdochium paspali* infection.
